# Scaling the production of bromine-76 and bromine-77 labeled poly-ADP-ribose-polymerase-targeted theranostics for preclinical use

**DOI:** 10.1186/s41181-026-00458-8

**Published:** 2026-06-01

**Authors:** Taylor R. Johnson, Hong Beom Lee, Yngve Guttormsen, Jason C. Mixdorf, Todd E. Barnhart, Jonathan W. Engle, Paul A. Ellison

**Affiliations:** 1 Department of Medical Physics, University of Wisconsin School of Medicine and Public Health, Madison, WI USA; 2 Department of Radiology, University of Wisconsin School of Medicine and Public Health, Madison, WI USA

**Keywords:** Radiobromine, Bromine-76, Bromine-77, Radionuclide production, Dry distillation, PARP inhibitor

## Abstract

**Background:**

Bromine-76, a positron emitting radionuclide for positron emission tomography (PET) imaging, and bromine-77, an Auger electron (Ae) emitting radionuclide, make a unique halogen theranostic pair. These isotopes have previously been used to synthesize a rucaparib-derived, poly-ADP-ribose polymerase inhibitor ([^76/77^Br]RD1). As [^77^Br]bromide activity in [^76/77^Br]RD1 radiosyntheses increased, coincident with solid target hardware changes in our facility, radiopharmaceutical yield became less reproducible. This work describes the modifications to this hardware, distillation procedures, and [^76/77^Br]bromide solution chemistry to enable reproducible production of preclinical quantities of [^76/77^Br]RD1.

**Results:**

Isotopically enriched cobalt selenide targets had production yields of 50.7 ± 8.7 MBq/µAh for bromine-76 and 14.5 ± 3.3 MBq/µAh for bromine-77, consistent with previous reports. Adoption of the GE PETtrace Solid Target Platform (STP) increased target backing diameter to 22 mm. Increasing dry distillation duration from 5 to 6 min to 8–9 min and furnace temperature from 1050 to 1055 °C released 94 ± 5% (*n* = 17) of activity, in agreement with previous smaller targets (92 ± 13%, *n* = 35). Rinsing glassware and loading the quaternary methyl ammonium (QMA) anion exchange cartridge in 1 mM sodium bicarbonate reduced [^76/77^Br]bromide elution volume from 2.9 to 0.9 mL. Across all distillations, when [^77^Br]bromide was isolated in less than 1 mL of QMA eluant, copper-mediated bromodeborylation of a BPin precursor molecule formed [^76/77^Br]RD1 with a radiochemical conversion of 98 ± 3% (*n* = 17) and overall radiopharmaceutical yield of 70 ± 10% (*n* = 15) following purification and final formulation. Up to 580 MBq of purified [^77^Br]RD1 have been produced with this system.

**Conclusion:**

The new target production technique made targets suitable for long-term use with the GE PETtrace STP. Following modifications to the distillation process, [^76/77^Br]bromide production yields from GE PETtrace STP targets agreed with those previously published. The changes described in this work not only aimed to maximize [^76/77^Br]bromide recovery after distillation, but also chemical reactivity in large-scale [^76/77^Br]RD1 radiopharmaceutical syntheses. This balancing act resulted in a slightly lower [^76/77^Br]bromide recovery yield but more consistently [^76/77^Br]RD1 product yield.

**Supplementary Information:**

The online version contains supplementary material available at 10.1186/s41181-026-00458-8.

## Background

Isotopes of bromine hold potential as both diagnostic and therapeutic radionuclides due to their unique decay properties and ability to form covalent bonds in small biological molecules, such as amino acids (Hanaoka et al. 2015), hormones (Zhou et al. 2008), nucleosides (Mercer et al. 1989), and derivatives of small molecule protein inhibitors (Lundkvist et al. 1999, Wilbur and Adam 2019). In particular, a diagnostic radiopharmaceutical labeled with bromine-76 (t_1/2_ = 16.2 h, positron decay branching ratio (BR_β+_) = 57%, BR_electron capture (ec)_ = 43%) for positron emission tomography- (PET-) based disease staging and patient selection could be paired with a therapeutic radiopharmaceutical labeled with bromine-77 (t_1/2_ = 57 h, BR_ec_ = 99.3%, BR_β+_ = 0.7%) forming a theranostic pair (ICRP 2008). The therapeutic emissions of bromine-77 are a cascade of 6–7 Auger electrons (Ae) (E_avg, Ae_ = 0.8 keV) with linear energy transfer of 4−26 keV/µm and subcellular (~ 0.1 μm) ranges (ICRP 2008, Bolcaen et al. 2023, Ku et al. 2019). The short ranges of Ae are cited to explain early reports of increased radiation damage when bromine-77 is incorporated into the DNA as [^77^Br]bromodeoxyuridine (vs. cytosol or extracellular locations) (Kassis et al. 1982, DeSombre et al. 1988). More recently, several bromine-77- and iodine-123-labeled Ae-emitting radiopharmaceuticals have targeted the poly-ADP ribose polymerase (PARP) class of nuclear DNA damage response proteins by derivatizing clinical small molecule PARP inhibitors (e.g. rucaparib) to direct the Ae-emitting payload to the nucleus (Filippi et al. 2024).

However, further exploration is currently limited by the ability to reliably produce sufficient quantities of radiobrominated PARP inhibitors. Current methods involve copper-mediated bromodeborylation of aryl boronic pinacol ester precursors, including the rucaparib derivative referred to in this work as [^77^Br]RD1 (also known as [^77^Br]Br-WC-DZ) (Ellison et al. 2020, Zhou et al. 2018, Sreekumar et al. 2023, Muzzioli et al. 2023, Mixdorf et al. 2023). In previously published investigations, typically only 1−100 MBq of activity were used, accounting for only a fraction of the ~ 500 MBq of bromine-77 produced from a routine 1 h, 30 µA irradiation (Zhou et al. 2018, Sreekumar et al. 2023, Muzzioli et al. 2023). However, scaling the synthesis to these higher activities preceded observation of reduced consistency and lower radiochemical conversion (RCC), with some reactions failing completely. Reclamation of radiobromide from a post-distillation anion exchange step also worsened, requiring larger volumes of base to elute the bromide, which resulted in lower RCCs to [^77^Br]RD1. For greater radiosynthesis reproducibility, the radiobromine distillation process needed adjustment, explored in this work.

Improvements to bromine-76 or bromine-77 radionuclide production methodologies, including the use of isotopically enriched cobalt selenide targets, the ARTMS QUANTM irradiation system (QIS), vertical dry distillation, and an anion exchange step for bromide volume reduction, were previously described (Ellison et al. 2020, Mixdorf et al. 2023). More recently, the University of Wisconsin transitioned to a GE PETtrace Solid Target Platform (STP). This shift required a larger diameter cyclotron target, which in turn prompted minor changes to the distillation apparatus components, heating time, and furnace temperature. Inclusion of these modifications served both to update to previous methods as well as describe choices to maintain radionuclide production and recovery yields as efforts to increase scale of [^76/77^Br]RD1 synthesis continued.

This work hypothesizes that modifications to radiobromine distillation processes and radiochemical methods can help increase consistent production of [^76^Br]RD1 and [^77^Br]RD1. Longitudinal data trends demonstrating reproducibility and sustainability of these radionuclide production and radiopharmaceutical synthetic procedures are also presented. These improvements aim to enable preclinical therapeutic efficacy studies of radiobrominated Ae-emitting radiopharmaceuticals.

## Methods

### Target production

Cobalt powder (Alfa Aesar, 1.6-micron, 99.8% metal basis) and isotopically enriched selenium (Isoflex, selenium-76 or selenium-77, Table S1) were added to a quartz ampule in a 1:1 atomic ratio, for a total of 240−360 mg. The ampule was evacuated, argon flushed, and sealed. A furnace (Thermolyne 10500 furnace or Carbolite Gero TF1 11/32/150) heated the ampule at 16 °C/min to 1100 °C, which was held for 1 h, and then the ampule was rapidly cooled in room temperature water. The ampule was broken open and the fused cobalt selenide ingot massed. The expected target dimensions were calculated using the cobalt selenide ingot mass, a cobalt selenide density of 7.65 g/cm^3^ (Lide 2004), and the diameter of the niobium backing pocket (9.5–11.4 mm). These dimensions informed the design of a graphite pellet press die set (Fig. S1a) and target tamper (Fig. S1b). The cobalt selenide ingot was positioned in the pressing die set before being inserted into a flat-bottomed quartz tube, with a quartz rod placed on the tamper within the sealed tube. Under flowing argon, the tube was lowered into a preheated furnace (1075 °C, Carbolite Gero, TF1 11/32/150, Fig. S2). The apparatus was heated for 7–12 min before the bell-shaped quartz tube was manually pressed on the quartz rod and tamper, and the quartz tube was removed to air cool. Upon reaching handleable temperature, the apparatus was disassembled completely. The flat, round disk was placed in a niobium target backing (2 mm thick, 19–22 mm diameter, depending on target platform, with a 1 mm deep pocket) with the target tamper on top (Fig. S1b), the apparatus reassembled, and the same heating and pressing process repeated. After water-cooling, the resulting cobalt selenide on niobium target was ready for cyclotron irradiation (Fig. S1c). Selenium-76 starting material was used to produce bromine-76, and selenium-77 to make bromine-77.

### Target irradiation

Before irradiation, the target and distillation apparatus were prepared and cleaned, with details in the supplemental materials. For irradiation, the cobalt-[^76^Se] or –[^77^Se]selenide (Co^76/77^Se) targets were loaded into one of two target delivery systems. In the ARTMS QIS, a 19 mm diameter target backing (originally designed for a manual cyclotron target system) was assembled into a titanium/aluminum target capsule (Fig. S3). The second target delivery system used was the STP (GE Healthcare). Before each production, the 22 mm diameter target was placed face-up in the aluminum backing (additional information in SI), covered with a 10.5 μm thick, 17.5 mm diameter tantalum foil, and assembled below the PEEK capsule cap (Fig. S4). Once assembled, the target capsules were pressure tested and sent to a specific port on University of Wisconsin-Madison’s GE Healthcare’s PETtrace cyclotron. The water-cooled target was bombarded with energy-degraded 12.5 or 13 MeV protons (for ARTMS QIS or GE STP, respectively) for 1–2 h at 30−35 µA. The STP target front face was cooled with flowing helium. After irradiation, the shuttle was retrieved and left for 40 min to decay bromine-77m (t_1/2_ = 4.3 min).

### Radiobromine distillation

**Fig. 1:**
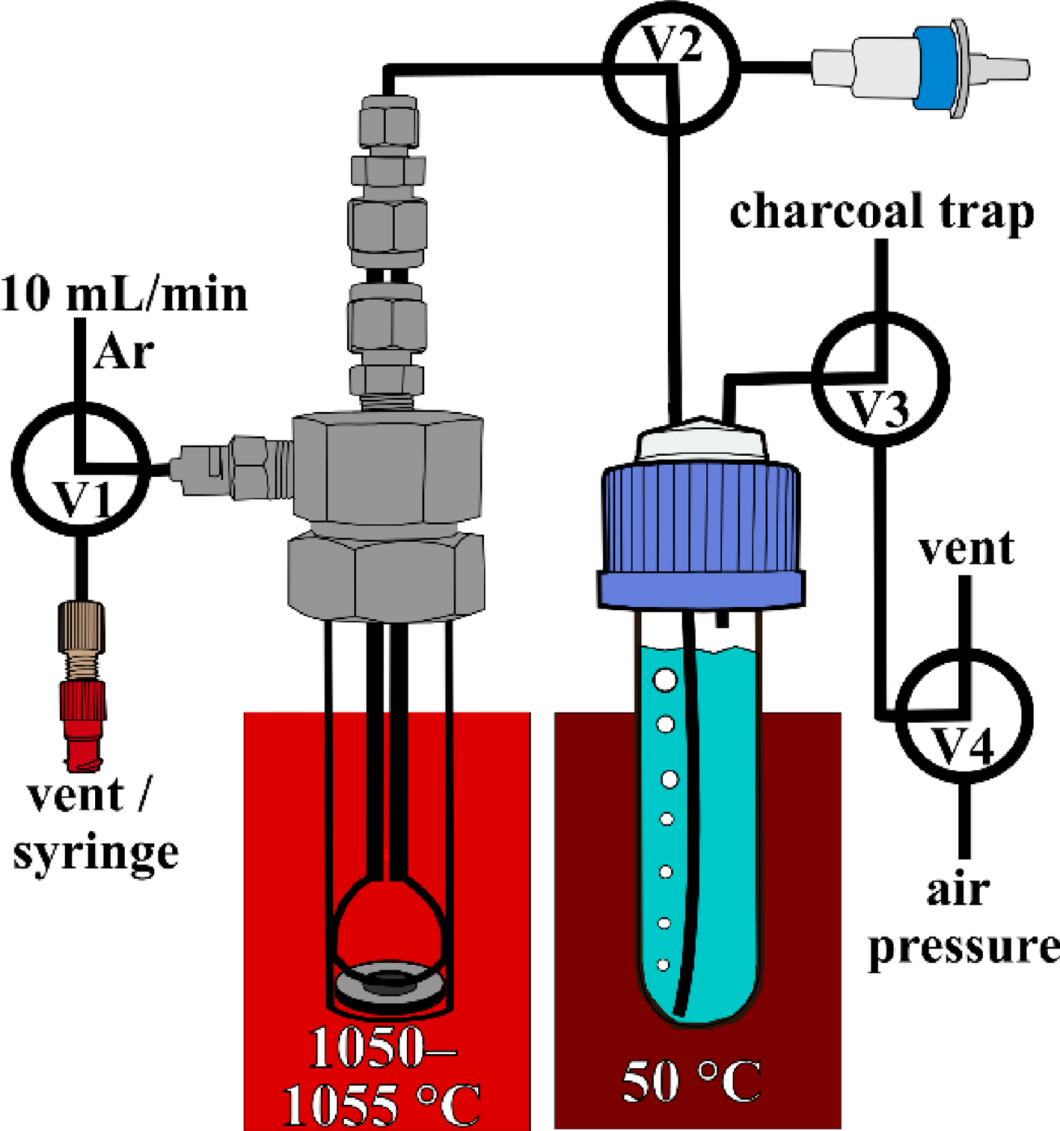
Furnace, valve, and tubing diagram for bromine-76 or bromine-77 dry distillation

The shuttle was disassembled and the target coin placed face up in a flat-bottomed quartz tube. This tube was connected to a system with 10 mL/min argon flow, 3.2 mm outer diameter PTFE tubing, three-way valves (V1, V3, V4: Swagelok SS-41GXS2; V2: Bürkert 6724), a liquid trap (35 mL of water or 1 mM sodium bicarbonate (NaHCO_3_), 50 °C), and charcoal trap (50 mL activated charcoal, EMD, 6–16 mesh) (Fig. 1). The tube was lowered into the tube furnace (originally preheated 1050 °C but raised to 1055 °C for 22 mm targets to improve bromide release) and allowed to heat for 5−7 or 6–9 min (for the 19–22 mm targets, respectively). Two 1 cm thallium-doped cesium iodide (CsI(Tl)) radiation detectors tracked the location of radioactivity in the system with data logged by National Instruments Labview (Da Silva et al. 2021). The detectors—lead-collimated on the base of the quartz tube in the furnace or the liquid trap—tracked radiobromine activity as it volatilized out of the target and transited to the liquid trap. Distillation was stopped when the rate of activity leaving the target slowed to near zero.

After the furnace radiation signal stabilized, the quartz tube was removed from the furnace and cooled in a room temperature water bath. The argon flow was temporarily increased from 10 mL/min to account for the lower pressure from rapidly cooled air within the tubing. Once cool, the apparatus was inverted and vented by actuating valves V1 and V3 (Fig. 1). A 60 mL syringe connected to V1 pulled the liquid from the trap to rinse the lines and bell-shaped quartz tube, then returned the rinse to the liquid trap with collected activity. This process was repeated until there was no longer a noticeable increase in liquid trap activity.

V2 was actuated to connect the liquid trap to a quaternary methylammonium (QMA) anion exchange cartridge (Sep-Pak Plus Light or Plus Short, Waters), prepared with 5 mL ethanol, 5 mL buffer solution (0.5 M NaHCO_3_, 0.5 M sodium carbonate (Na_2_CO_3_), or a 50/50 mixture), and 10 mL of water. V4 was then actuated to push dissolved bromide from the trap through the QMA cartridge into a waste tube, trapping radiobromide on the cartridge resin. An additional 10 mL water rinsed the cartridge. A dose calibrator (Capintec CRC-15R, setting #690/2 for bromine-76 or #120 for bromine-77) measured the activity that had adsorbed to the cartridge and that had passed through to the waste tube, then the cartridge was eluted with 0.8–7.4 mL 0.1 M dimethylamine (DMA, Sigma Aldrich, 2 M in methanol) in 50/50 acetonitrile/water. A heat block dried the eluate at 130 °C under argon flow.

After the distillation, the apparatus was disassembled, and each component’s activity was measured with the dose calibrator. The target coin, now containing arsenic-74 (^77^Se(p,α)^74^As, t_1/2_ = 17.8 d), molybdenum-93m (^93^Nb(p,n)^93m^Mo, t_1/2_ = 6.85 h), niobium-92m (^93^Nb(p,pn)^92m^Nb, t_1/2_ = 10.2 d), cobalt-58g (^59^Co(p,pn)^58g^Co, t_1/2_ = 70.9 d), and bromine-76/bromine-77, was assayed by high purity germanium (HPGe) spectroscopy (full width at half maximum 1333 keV = 1.6 keV, Canberra Inc., Meriden, CT, USA). HPGe efficiency was calibrated using americium-241, cesium-137, europium-152, barium-133, and cobalt-60 (Amersham International PLC gamma reference sources, set 1718) measured at 60 cm from the face of the detector. A sample of the QMA eluate was also routinely assayed by HPGe to determine radionuclidic purity of captured bromine. Characteristic gamma peaks were used to quantify bromine-77 (239.0 and 520.6 keV) and bromine-76 (559.1 and 657.9 keV).

Chemical purity was assessed with a styrene-divinyl benzene polymer resin functionalized with cation exchange groups preloaded with silver counterions (IC-Ag cartridge, Grace Alltech Maxi-Clean). A sample of dried radiobromine was diluted into 10 mL water and passed through the column, reacting bromine with silver ions to form silver bromide or silver bromate. As silver bromide is insoluble in water and silver bromate very soluble, any activity remaining on the column exists as bromide (Dhillon and Statler 2012). Radiochemical purity was calculated by dividing the activity trapped on the column by the sum of activity in the column and its water rinse.

### Radiopharmaceutical synthesis

**Fig. 2:**
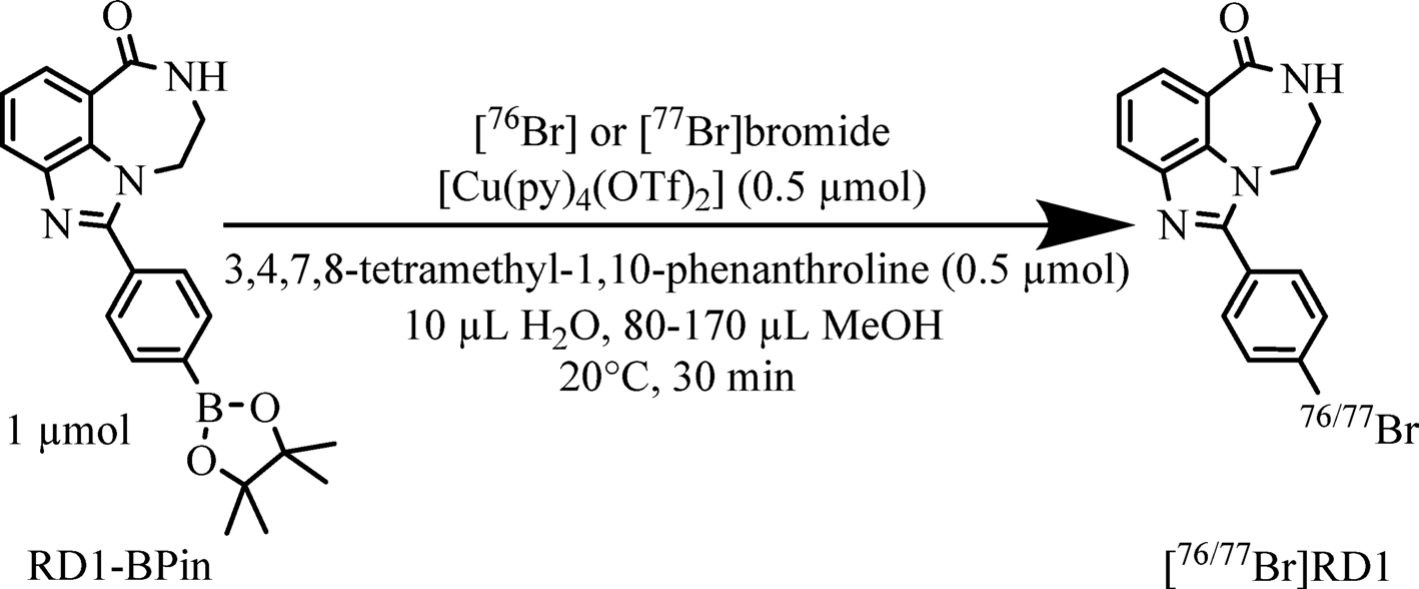
Reaction scheme for radiobrominating RD1-BPin into [^76^Br]RD1 or [^77^Br]RD1

Besides the different Co^76/77^Se targets used for cyclotron irradiation, no differences were made between the radiopharmaceutical synthesis and purification methodologies for [^76^Br]RD1 and [^77^Br]RD1. [^76/77^Br]RD1 is used throughout this manuscript to generally describe RD1 labeled with either bromine-76 or bromine-77. The boronic acid pinacol ester (BPin) precursor for the rucaparib-derived radiopharmaceutical (RD1-BPin) was synthesized in-house adapting literature procedures (Reilly et al. 2018) (see SI and Figs. S5–S9 for additional details). RD1-Bpin (1 µmol, 0.4 ± 0.1 mg) was added to a borosilicate pulled-point vial (1.1 mL, SUN-Sri) with 80−170 µL of methanol and vortexed until fully dissolved. 3,4,7,8-tetramethyl-1,10-phenanthroline (4.6 µmol, 1.1 mg) and tetrakis(pyridine)copper(II) triflate (4.6 µmol, 3.1 mg) were likewise dissolved in 100 µL methanol and vortexed until a uniform royal blue, forming the “copper-ligand” (Cu-L) solution. 10 µL water, 10 µL Cu-L (0.5 µmol), all the BPin solution, and a small stir bar were added to the vial of dried radiobromine (25−790 MBq bromine-77 or 140−520 MBq bromine-76). The mixture reacted at room temperature under constant agitation for 30 min (Fig. 2).

At completion, the reaction was diluted and vial rinsed with water (5 mL). The diluted reaction mixture was passed through an octadecyl- (C18-) cartridge (Sep-Pak Plus Light C18 Cartridges, Waters) prepared with 5 mL ethanol and 10 mL water, then rinsed with 10 mL of water. Radiochemical conversion was determined by dividing the activity trapped on the C18 cartridge by the total activity loaded onto the cartridge, as determined by dose calibrator measurements. Ethanol (0.5 mL) eluted the crude product off the C18 cartridge, then 0.5 mL of water rinsed the cartridge and diluted the product for purification via semi-preparative high performance liquid chromatography (HPLC).

An HPLC pump (Beckman 110B Solvent Delivery Module) flowed 70/30 0.1 M ammonium formate and acetonitrile (acidified with concentrated hydrochloric acid to 0.1% (v/v)) through a Phenomenex, Kinetex 5 μm XB-C18 100 Å, 250 × 10 mm column at 4 mL/min. After the crude product was injected, Labview logged the 254 nm UV absorbance (Knauer, Smartline 200) and the radioactivity (Carroll-Ramsey Instruments, model 105 S) of the column effluent. The purified radioactive product peak eluting around 13 min was collected into a gross dilution bottle prefilled with water (35 mL), which was then transferred through a second C18 cartridge (prepared as above) via air pressure (40−80 kPa). The C18 cartridge was rinsed with water (10 mL) then eluted through a sterile filter (13 mm syringe filter, Whatman, 0.2 μm PSU filter media) with 0.5–0.8 mL ethanol into a sterilized polypropylene tube. Argon gas flow dried the final, radiobrominated PARP inhibitor derivative over the course of an hour, with final activity measured via dose calibrator. Further use typically rehydrated the product in phosphate buffered saline (PBS) or sterile saline at desired activity concentrations for radiopharmaceutical evaluation in vitro and in vivo. Between productions, the semi-preparative HPLC was cleaned and stored in 65% acetonitrile/35% water.

Analytical HPLC (Agilent 1260 Infinity II) quantified the molar activity (A_m_) and radiochemical purity of [^76/77^Br]RD1 after final formulation. The radiation detection system consisted of a CsI(Tl) scintillator (1 × 1 cm, Hilger Crystals Ltd., Kent, UK) coupled to a photomultiplier tube (E849-35, Hamamatsu Photonics, Hamamatsu City, Japan) powered and processed with bench-top electronics (925-SCINT, Ortec, Oak Ridge, TN, USA), with electronic analog pulse converted to voltage (Model 106, Lawson Labs Inc., Malvern, PA, USA) and logged using an Agilent 1200 universal interface box. Samples (20 µL) were injected onto the column (Phenomenex C18, 2.6 µ, 100 Å) and run at 1 mL/min using a linear gradient method of 0.1 M triethylamine (TEA) or 0.1% trifluoroacetic acid (TFA) and acetonitrile (Table S2). Further details on establishing a [^nat^Br]RD1 calibration curve and measuring the radiochemical purity (RCP) and A_m_ can be found in the supplemental materials.

## Results

### Target production and irradiation

Physical properties of two cobalt [^76^Se]selenide (Co^76^Se) and six cobalt [^77^Se]selenide (Co^77^Se) targets are summarized in Table 1. While cobalt selenide thickness is challenging to measure once in the niobium backing due to its rough texture, it melded well with the backing with minimal spillover losses when utilizing the ingot to disk to target making procedure. From weighing the powder to a final target, cobalt selenide mass loss was 6 ± 7% (*n* = 3) for previous direct fused cobalt selenide pressing methods, and 5 ± 3% (*n* = 4) for methods using the intermediate disk step, no significant difference. Dropping the target coin slightly above (~10 cm) the benchtop resulted in no damage to the target, suggesting resistance to the cobalt selenide shattering or being detached from the backing upon small physical impacts that may occur with target handling.

**Table 1:** Summary of cobalt selenide targets used in this work

Compound	Starting mass	Mass thickness	Estimated thickness	Diameter	Irradiation system
Co^76^Se	488 mg	480 mg/cm^2^	630 μm	11.4 mm	ARTMS QIS
Co^76^Se	225 mg	290 mg/cm^2^	380 μm	9.9 mm	GE STP
Co^77^Se	349 mg	350 mg/cm^2^	450 μm	11.4 mm	ARTMS QIS
Co^77^Se	310 mg	400 mg/cm^2^	530 μm	9.9 mm	ARTMS QIS
Co^77^Se	224 mg	290 mg/cm^2^	380 μm	9.9 mm	GE STP
Co^77^Se	227 mg	300 mg/cm^2^	390 μm	9.9 mm	GE STP
Co^77^Se	220 mg	290 mg/cm^2^	370 μm	9.9 mm	GE STP

Irradiation yields were determined by summing recovered activity, non-recoverable distilled activity (e.g. on glassware), and residual activity in the target coin, then dividing by the irradiation charge. Production yields for bromine-76 were 53.3 ± 7.0 MBq/µAh (*n* = 7) for 11.4 mm inner diameter targets and 44.9 ± 10.8 MBq/µAh (*n* = 3) for 9.9 mm inner diameter targets. For bromine-77, production yields were 13.9 ± 2.8 MBq/µAh for 11.4 mm (*n* = 21) inner diameter targets and 14.9 ± 3.6 MBq/µAh (*n* = 38) for 9.9 mm targets. For both isotopes, the production yields were not significantly different between the 9.9 mm and 11.4 mm inner diameter targets.

Through the lifespan of each target, production yields generally decreased after each distillation, with an average decrease of 2 ± 3% (*n* = 4 targets) for bromine-77 and ~ 2% (*n* = 2 targets) for bromine-76 (Fig. S10). Overall, these targets tolerated the beam well, with the exception of the 530 μm (400 mg/cm^2^) cobalt [^77^Se]selenide target, which was excluded from this analysis. This target initially had high production yield of 21.9 MBq/µAh but saw an average yield loss of 10 ± 3% (*n* = 6) with each distillation. During irradiation, water cooling was insufficient, leading to the cobalt selenide darkening and distorting from the beam, potentially because of poor contact of the cobalt selenide with the niobium backing.

### Radiobromine distillation

Comparing the distillation yield for the 19 mm and 22 mm targets after a ~ 6 min distillation at 1050 °C, the released activity was 92 ± 13% (*n* = 35) and 65 ± 34% (*n* = 3), respectively, with one 22 mm target distillation releasing only 26% of the produced activity. By increasing heating duration to 8–9 min for the larger targets, distilled activity changed to 86 ± 13% (*n* = 15). However, one longer distillation removed only 66% of the activity from the target, prompting the increase in furnace temperature to 1055 °C. This led to an activity release of 94 ± 5% (*n* = 17), in alignment with the yields from the smaller targets. With these changes, all bromine-77 distillations have had at least 85% of the activity released (*n* = 16). Averaging across all distillations, 90 ± 14% (*n* = 70) of the produced bromine was removed from the target. After four or five rinses of the glassware with heated water or 1 mM sodium bicarbonate (Fig. S11), 68 ± 17% (*n* = 70) of total produced activity was recovered (75 ± 14% of the activity that was distilled out of the target).

The interaction of the distilled bromide with the QMA cartridge is highly sensitive not only to the QMA cartridge preparation, thoroughly explored in (16,21), but also to the loading solution pH. The challenge arose where larger volumes of DMA were needed to elute the QMA (also found in (22)). Instead of changing the elution solution, which could affect downstream processing, the pH of the load and preconditioning solution were modified using the same carbonate anion, detailed in Table 2. Across all distillations and solution types, 90 ± 17% of the bromide remained on the cartridge after loading the activity and rinsing. The pH of the solutions passing through the QMA cartridge during preparation and loading influenced its ability to trap activity, with high basicity obstructing bromide anion exchange. QMA preparation using sodium carbonate (pH ~11) or a mix of sodium carbonate and sodium bicarbonate (pH 10-11) lead to only 53 and 47% trapped activity (Table 2, bottom), respectively. Additionally, in one distillation, the glassware was improperly cleaned, leaving sodium hydroxide residue remaining in the glassware during the distillation (pH later measured at 10), resulting in only 2% activity adsorbed onto the QMA.

**Table 2:** QMA preconditioning and liquid trap solutions with their effect on activity trapping ability and elution volume with optimal conditions in bold

Overall pH	Precondition Buffer	Loading Solution	Percent Trapped	Elution	Method reference
Lower pH loading solution	0.5 M NaHCO_3_ (pH ~ 8.3)	H_2_O (pH ~ 4.5)	94 ± 5% (n = 9)	93 ± 6% in 2.9 ± 2.2 mL DMA (n = 9)	Mixdorf et al. (2023)
**Moderate pH**	**0.5 M NaHCO**_**3**_ ** (pH ~ 8.3)**	**1 mM NaHCO**_**3**_ ** (pH ~ 5)**	**87 ± 12% (n = 23)**	**93 ± 3% in 0.87 ± 0.13 mL DMA (n = 20)**	**This work**
Higher pH precondition	0.5 M Na_2_CO_3_ (pH ~ 11)	1 mM NaHCO_3_ (pH ~ 5)	~ 50%	~ 90% in 0.9 mL DMA	This work

Too low of a pH could also influence the ability to remove activity from the QMA cartridge, although the evidence for this is less conclusive. Examining the subset of distillations made from 22 mm targets, when activity was loaded onto the QMA cartridge in deionized water (pH ~ 4.5), it took between 1.2 and 7.4 mL of DMA solution to release at least 80% of the activity from the cartridge (*n* = 9, Table 2, bottom). However, when loaded on the QMA in 1 mM sodium bicarbonate (pH ~ 5), the volume required to elute at least 90% of the radiobromide was less than 1 mL (*n* = 20, Table 2 middle). The slight increase in pH changing from the deionized water to the 1 mM sodium bicarbonate (pH 5) could have provided a buffer against the pH fluctuations of the sodium bicarbonate QMA preparation solution to maintain sufficiently basic load and elution conditions.

After the distillation, residual target activity was measured via HPGe spectrometry. While radiobromine was quantified as part of determining production yields, cobalt-58 g (810.8 keV), niobium-92 m (634.5 keV), and arsenic-74 (595.8 keV) peaks were always present in in total quantities < 6 MBq. Based on the HPGe measurement of a small sample of the QMA elution, these radionuclides remain in the coin. Bromine-77 was produced with > 98.5% radionuclidic purity at the end of bombardment (EoB), with average purity of 99.5 ± 0.4% (*n* = 14). Bromine-76 was the only detected radionuclidic impurity. Chemical purity tests conducted using the IC-Ag columns found > 96% of the produced bromine-77 in bromide form, with an average chemical purity of 99.1 ± 1.0% (*n* = 14).

### Radiopharmaceutical synthesis

**Fig. 3:**
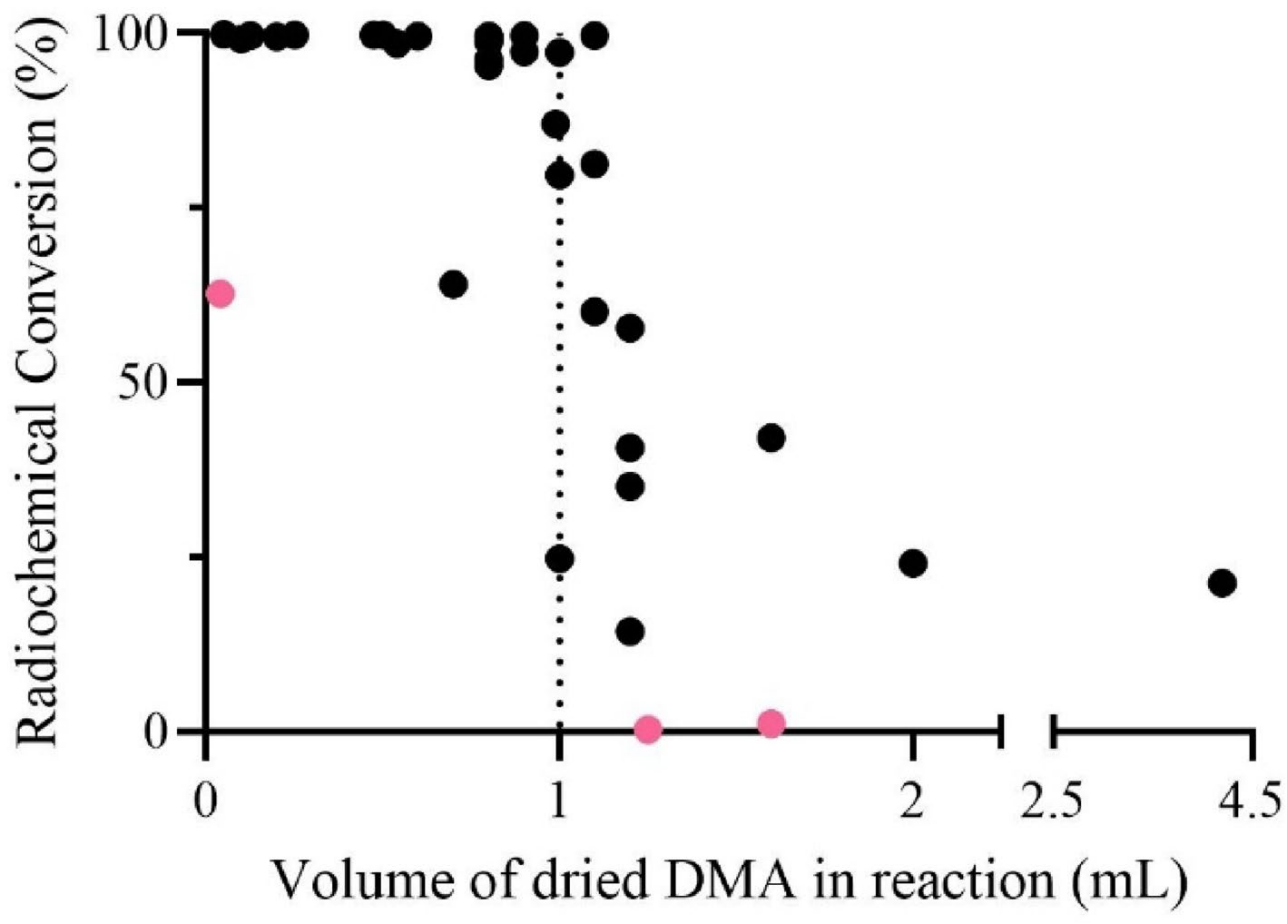
Plot displaying varying [^77^Br]RD1 RCC as a function of the volume of DMA eluted from the QMA that was dried and used in the reaction. Points displayed in pink represent reactions that used radiobromine solutions that dried pink

The radiobromodeborylation reaction formed [^76^Br]RD1 or [^77^Br]RD1 with an overall RCC of 77 ± 32% (*n* = 32). However, when reactions were scaled up to include greater amounts of radiobromide, a trend formed where reactions using [^77^Br]bromide dried from a larger volume of DMA or with pink or brown residue resulted in lower RCC (Fig. 3). When elution volumes were kept below 1 mL with no pink residue visible, reaction yields were 98 ± 3% (*n* = 17), as compared to 21 ± 36% (*n* = 3) for reactions using [^77^Br]bromide that dried pink or 57 ± 26% (*n* = 11) for reaction using [^77^Br]bromide that dried colorless from DMA elutions of ≥ 1 mL. Once this trend became apparent, if an elution fraction dried pink, the activity was reconstituted in water or 1 mM sodium bicarbonate and passed through another preconditioned QMA. Average trapping on this second QMA was 99%, with the 0.9 mL DMA elution drying down clear (*n* = 3).

**Fig. 4:**
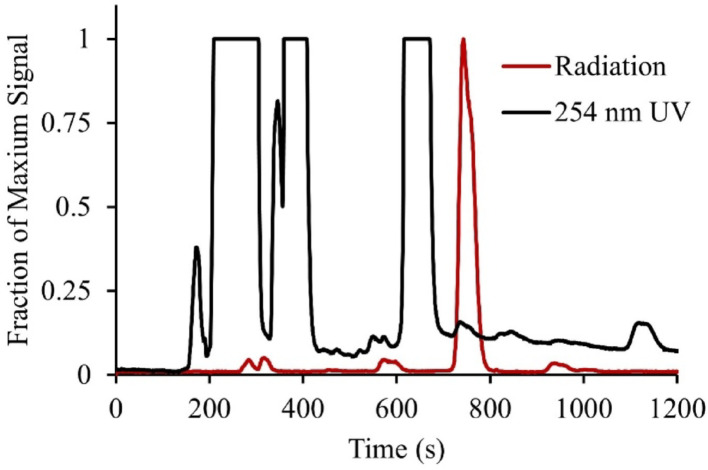
Example semi-preparative HPLC chromatogram for [^77^Br]RD1 purification with UV and radiation detector signals normalized to respective maximum value signal. The [^77^Br]RD1 eluted between 700 and 800 s

The semi-preparative HPLC effectively separated the radiopharmaceutical product from other compounds within the crude product mixture (injected in an ethanol/water mixture), with the [^76/77^Br]RD1 collected at around 790 s (Fig. 4). Other absorbance peaks in the chromatogram could potentially originate from unreacted pinacol ester precursor, the phenanthroline ligand, and a protodeborylation product known to form in copper-mediated reactions (Kaur et al. 2025). Reformulation of the purified radiopharmaceutical was high yielding and effective with 98% recovery from the second C18 cartridge in ethanol. Subsequent evaporation of the [^76/77^Br]RD1 product took 1–3 h under argon flow. Excluding the first drop (~ 50 µL) of the ethanol eluate reduced the dry time of the final product without loss of activity. Final non-decay corrected activity yield was 58 ± 21% (*n* = 30) across all reactions but 70 ± 10% (*n* = 15) for [^77^Br]RD1 reactions using bromide dried clear from less than 1 mL DMA.

Fitting the resultant mass peak to the standard calibration curve returned the molar amount of the sample (Fig. S12), which when adjusted by the injected activity and the percent free bromide, resulted in a radiopharmaceutical molar activity (A_m_) in units of GBq/µmol. Between different productions, the A_m_ varied significantly and was proportional to overall formulated [^77^Br]RD1 activity (Fig. S13). For synthesis resulting in > 80 MBq of [^77^Br]RD1, A_m_ was 220 ± 110 GBq/µmol (*n* = 10). When measured within 20 h of end of synthesis, [^76/77^Br]RD1 had 99.3 ± 1.0% (*n* = 29) radiochemical purity.

## Discussion

The primary goal of this work was to update and expand upon previous reports of the cyclotron production of bromine-77/76 and synthesis of rucaparib-derived radiobrominated pharmaceuticals (Ellison et al. 2020), as many minor changes have been implemented in efforts to produce quantities suitable for larger scale preclinical biological evaluation. The greatest changes occurred due to the adaptation of cyclotron solid target from one automated solid target system to another, requiring a change in target size that affected target preparation, the distillation apparatus, furnace settings, distillation time, rinsing procedure, and QMA elution. Increased biological testing also required process modifications to ensure that long-term performances of the targets could withstand longer irradiations and that the distillation procedures would reproducibly isolate radiobromine in a state resulting in good radiochemical conversion rates.

Enriched cobalt selenide targets have proven resilient to multi-hour, 30−35 µA irradiations by 13 MeV protons. However, with pink residue on distillation glassware and the gradual decrease in production yields over time, minor selenium loss occurs during each production and distillation process. Exact selenium mass losses are challenging to track for the 22 mm diameter targets due to flaking of the niobium backing during the distillation process and sanding of the niobium during target preparation to ensure good seal with the o-ring (see supplemental materials). For example, over the course of 13 irradiations (totaling 948 µAh) and 14 distillations, one of the targets had lost 276.2 mg, more than the starting cobalt selenide mass of 227.1 mg, yet still retained sufficiently high production yield (17.6 MBq/µAh) with a recent irradiation. As it is physically impossible for this mass loss to come solely from the cobalt selenide, tracking target mass over time does not accurately represent cobalt selenide target material losses.

Greater evidence of the durability and reusable nature of these targets comes in the form of production yields. When compared with the previously reported yields of 50 MBq/µAh (at 13 MeV) from bromine-76 and 13.1 MBq/µAh (at 12 MeV) for bromine-77 (Ellison et al. 2020), the production yields of 50.7 and 14.5 MBq/µAh align extremely well. Target fabrication methods created a strong bond between the cobalt selenide and niobium backing material that, coupled with the dry distillation method, allowed for repeated target use. Additionally, by creating a cobalt selenide disk using the graphite pellet press die before pressing it into the backing, the cobalt selenide is already evenly covering the pocket and must simply adhere to the backing to complete target construction. Another study using the previous construction technique for an enriched cobalt [^76^Se]selenide target (24 mm overall diameter, 12 mm pocket diameter) produced an even higher yield of ~ 72 MBq/µAh (Franke et al. 2024), though the discrepancy was likely due to a different beam spot from the IBA Cyclone 18/9 cyclotron used in that work. Because the beam spot of the GE PETtrace cyclotron is larger than both the 9.9 and 11.4 mm diameter cobalt selenide targets (Ellison et al. 2020), the larger diameter target were hypothesized to result in a higher production yield. However, as the reported yields were averaged over many irradiation/distillation cycles with production yields degrading with continued use, no significant differences were observed between the lifetime averaged radiobromine production yields from 9.9 to 11.4 mm diameter cobalt selenide targets.

Overall, production yields were predictable with only about 2% decrease with each distillation. Variation between distillations was likely due to activity quantification challenges (measuring long, oddly shaped, or self-shielding objects used in the distillation) and fluctuations in transverse beam intensity distribution with cyclotron magnet temperature, radiofrequency tuning, ion source rebuilds, central region geometry, extractor foil condition, and target/degrader/irradiation station geometries. However, one thicker (530 μm, 19 mm outer diameter, ARTMS) bromine-77 target saw rapid yield loss (~ 10%) with each distillation. Based on the physical appearance of the target after multiple irradiations where it looked like an oval hole had been burned into the cobalt selenide, it was hypothesized that for the thicker target geometry produced a thermal contact or morphology that diminished water cooling efficacy. In comparison, the bromine-76 19 mm outer diameter (ARTMS) target had a thickness of 630 μm but tolerated the beam well, receiving about the same cumulative current (260 µAh vs. 280 µAh for the bromine-77 target). The construction of the thicker bromine-77 target used the direct ingot to target method, but because all targets made with the ingot-disk-target method were thinner (~ 380 μm), differences in yield decreases over time as a function of target making process are obscured by different target thicknesses.

The other aspect of radiobromine production, the distillation, is a challenging balancing act. The variables of furnace temperature, length of time in furnace, time to rinse after quenching, argon flow rate, liquid trap composition and temperature, choice of solid phase extraction cartridge, QMA preconditioning method, and even the cleaning procedure for the glassware and tubing (see supplemental materials) can all affect the success of a distillation. Not only that, but success is measured in various ways, such as the amount of activity removed from the target, the purity of collected activity from the target material, and the fraction of total produced activity in the final QMA eluant. In that regard, this distillation procedure removing 90 ± 15% or the radiobromine from the target is in line with other reported separations (Ellison et al. 2020; Franke et al. 2024; Hassan et al. 2017; Breunig et al. 2015; Vaalburg et al. 1985). Increasing furnace dwell time even more to 20 min could be used to increase removal of radiobromide from the target, as implemented in other dry distillation procedures (Breunig et al. 2015; Vaalburg et al. 1985; Ellison et al. 2017) and when moving to the GE STP system. The larger (22 mm niobium diameter) targets of the GE STP system necessitated a larger glassware and distillation apparatus, increasing the time and energy input required to heat the target to distillation level temperatures, requiring longer heat times and furnace temperatures.

Other potential changes have been considered and implemented to ensure high distillation success in all definitions. One implemented modification included the exchange of the outer, flat-bottomed quartz tube containing the target with a clean empty one between heating and rinsing stages of the distillation. This process was implemented to avoid contamination by the flakes that arise from the niobium backing during heating, a new issue that occurred only upon use of the larger targets. Many previous studies also implemented a sharper temperature gradient along the path of activity to encourage bromide deposition in certain locations, including previously with an ice bath (Ellison et al. 2020), but activity typically deposited within the glassware just above the furnace, making such measures unnecessary.

The largest change applied for the distillation procedure was using 1 mM sodium bicarbonate instead of water to rinse the glassware and lines, lowering necessary QMA elution volume. While DMA elution has presented consistency challenges and other groups have been unable to elute with it at all (Franke et al. 2024), it provides both the basicity to strip the activity from the QMA and the volatility to dry rapidly. However, when the activity was challenging to elute fully and large amounts of DMA were used, later reactions using the dried product had poor radiochemical conversions in subsequent bromodeborylation reactions. As high base content causes low radiochemical conversion in copper mediated radiofluorination of aryl boronates (Zlatopolskiy et al. 2015) and is detrimental to the copper mediated radioiodination of RD1-Bpin (Zhou et al. 2023), it is possible that the DMA residue itself disrupted the reactions. Using activity dried from volumes above 1 mL DMA resulted in poor RCC (57 ± 26%, *n* = 11), supporting this conclusion. However, a reaction eluted with small volumes of DMA that dried pink also had poorer than expected RCC, implying there could be a secondary route of reaction failure. With this, it was hypothesized that the DMA eluted trace amounts of selenium from the QMA cartridge after the bromide, overlapping with the trailing end of the activity. Greater volumes of DMA could release more of the captured selenium, which could result in a pink residue and disrupt the reaction. While a detailed investigation of the pink residue’s identity and its impact on subsequent radiobromination reactions have been left for future work, dilution and QMA repurification of the radiobromide was shown to be a viable remediation strategy. Overall, with the change of loading solution to 1 mM sodium bicarbonate, the percentage of radiobromide collected in the liquid trap that traps on the QMA was reduced, but downstream bromodeborylation RCC and consistency improved.

Calculated molar activity varied significantly between different syntheses with high values measured for successful reactions. Some variation could be accounted for by the range of activities used in reactions (25−790 MBq), as molar activity was positively correlated to final formulated activity. This correlation could indicate that the RD1-BPin precursor utilized in this work contained a small (~ nmol scale, 0.1%) [^nat^Br]RD1 impurity or that the reaction mixture contained radioactivity-invariant nmol scale non-radioactive [^nat^Br]bromide contaminant, potentially from the QMA preparation/elution solution, copper/ligand mixture or reaction solvent. The molar activities measured in this work were suitable for extensive preclinical evaluation of [^77^Br]RD1 and [^76^Br]RD1.

The [^76^Br]RD1 and [^77^Br]RD1 reactions were successfully scaled up for use in biological studies. Some of these reactions included more than 700 MBq of [^77^Br]bromide (resulting in a final production of 580 MBq [^77^Br]RD1) and still had high radiochemical conversion, allowing for preclinical therapeutic efficacy studies. With studies of this size, being able to get to most activity out of a production while not compromising radiopharmaceutical synthesis yields was essential, necessitating all the procedural changes detailed above.

## Conclusion

This manuscript reports changes to target construction, dry distillation, and radiobromide processing toward the production of bromine-76- or bromine77-labeled PARP-targeted radiopharmaceuticals. Radiobromine can be produced and isolated with high yields in a reliable, cost-effective way that allows for incorporation into small molecule vectors. This work describes resilient accelerator target synthesis and effective capture of volatilized bromine used for radiopharmaceutical synthesis of preclinical-scale biological evaluations of theranostic agents.

## Supplementary Information

Below is the link to the electronic supplementary material.

Supplementary Material 1

## Data Availability

The datasets used and/or analyzed during the current study are available from the corresponding author on reasonable request.

## Abbreviations

BR: Branching ratio
EC: Electron capture
PET: Positron emission tomography
Ae: Auger electrons
DNA: Deoxyribonucleic acid
PARP: Poly-ADP-ribose polymerase
RD1: Rucaparib derivative 1
RCC: Radiochemical conversion
QIS: QUANTM irradiation system
STP: Solid target platform
PEEK: Polyether ether ketone
PTFE: Polytetrafluoroethylene
QMA: Quaternary methylammonium
DMA: Dimethylamine
HPGe: High purity germanium
BPin: Bis(pinacolato)diboron
HPLC: High performance liquid chromatography
TEA: Triethylamine
TFA: Trifluoroacetic acid
UV: Ultraviolet
A_m_: Molar activity
RCP: Radiochemical purity
EoB: End of bombardment (time)
