# Supplementary material for: Scaling the production of bromine-76 and bromine-77 labeled poly-ADP-ribose-polymerase-targeted theranostics for preclinical use
Source: EJNMMI Radiopharm Chem. 2026 Jun 1;11:52. doi: 10.1186/s41181-026-00458-8 (PMC13437825; doi:10.1186/s41181-026-00458-8)

Supporting Information:

Scaling the production of bromine-76 and bromine-77 labeled poly-ADP-ribose-polymerase-targeted theranostics for preclinical use

Taylor R. Johnson[a], Hong Beom Lee[a], Yngve Guttormsen[a], Jason C. Mixdorf[a], Todd E. Barnhart[a], Jonathan W. Engle[a,b], Paul A. Ellison[a]

[a]Department of Medical Physics, University of Wisconsin, Madison, WI and [b]Department of Radiology, University of Wisconsin, Madison, WI.

Table of Contents

SUPPLEMENTAL TABLE 1……………………………………………………………………….2

SUPPLEMENTAL FIGURE S1………………………………………………………………….2

SUPPLEMENTAL FIGURE S2………………………………………………………………….3

DISTILLATION PREPARATION PROCEDURE……………………………………………..4

SUPPLEMENTAL FIGURE S3………………………………………………………………….5

SUPPLEMENTAL FIGURE S4………………………………………………………………….6

SYNTHETIC AND ANALYTICAL CHEMISTRY DETAILS………………………………..7

SUPPLEMENTAL FIGURE S5………………………………………………………………….7

SUPPLEMENTAL FIGURE S6………………………………………………………………….8

SUPPLEMENTAL FIGURE S7………………………………………………………………….9

SUPPLEMENTAL FIGURE S8………………………………………………………………...10

SUPPLEMENTAL FIGURE S9………………………………………………………………...11

SUPPLEMENTAL TABLE 2…………………………………………………………………...12

MOLAR ACTIVITY DETERMINATION METHODS………………………………………12

SUPPLEMENTAL FIGURE S10………………………………………………………………..13

SUPPLEMENTAL FIGURE S11………………………………………………………………..14

SUPPLEMENTAL FIGURE S12………………………………………………………………..15

SUPPLEMENTAL FIGURE S13………………………………………………………………..16

Supplemental Table 1. Isotopic abundances of isotopically enriched selenium powders (Isoflex USA).

	Selenium Isotopic Content (%)					
	^{74}Se	^{76}Se	^{77}Se	^{78}Se	^{80}Se	^{82}Se
^{76}Se Powder	<0.05	99.80 (±0.1)	0.2 (±0.1)	<0.05	<0.05	<0.05
^{77}Se Powder	0.02	0.14	99.66	0.09	0.05	0.04

Supplemental Figure S1. a) graphite crucible and crucible tamper with cobalt selenide (CoSe) ingot, b) pocketed niobium backing with flattened cobalt selenide disk and matching target tamper, and c) the final constructed cobalt selenide target.

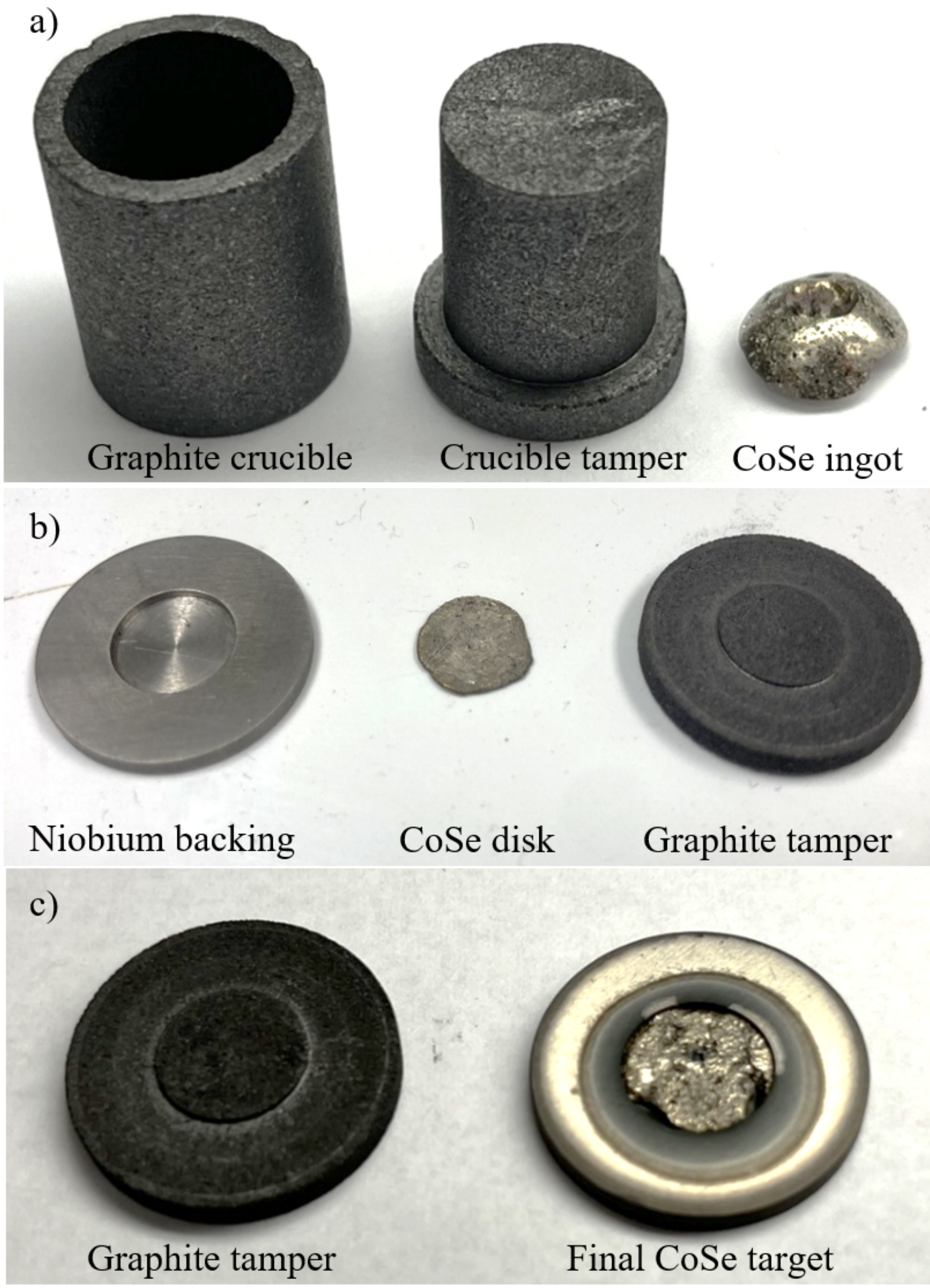

Supplemental Figure S2. Furnace apparatus for constructing targets, including the furnace, tube for holding the target coin or tamper, and a 35 g quartz rod for applying consistent weight while heating.

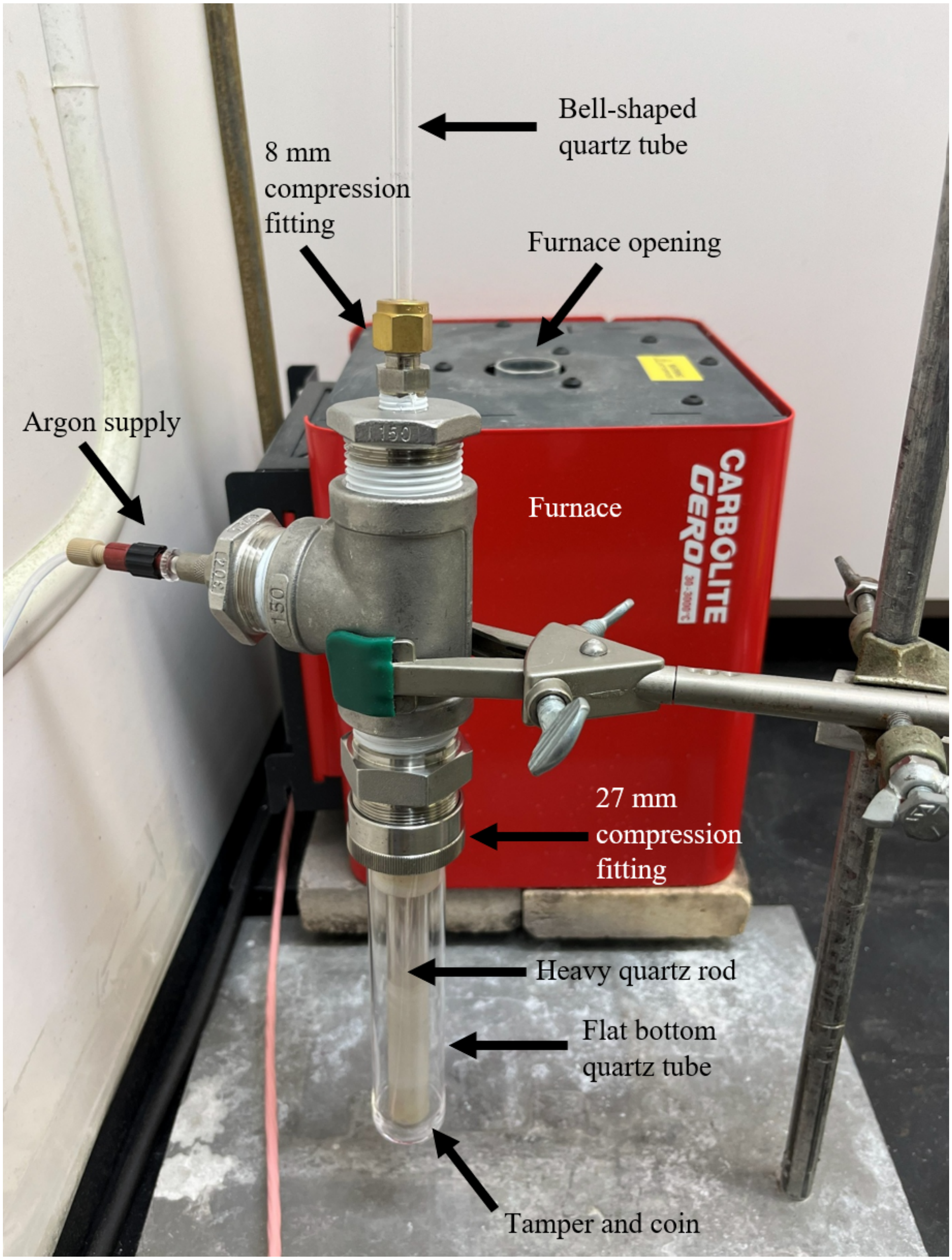

Distillation Preparation Procedure

Purpose: To establish reproducible distillation results via consistent cleaning, processing, and preparation of reusable distillation materials

Methods:

1. **Clean distillation glassware.** Rinse bell-shaped quartz tube and flat-bottomed quartz tube with concentrated nitric acid while heating with a heat gun (Varitemp Heat Gun, model VT-750C, Master Appliance Corp.) until all pink and black residue is removed. Rinse with water, followed by rinsing with concentrated sodium hydroxide while heating until all remaining visible contamination is removed. Scrub quartz glassware, resuable vials, and liquid trap with aqueous detergent solution (Powdered Precision Cleaner, Alconox Inc), rinsing with tap water then deionized water, and dry in an oven (VWE Scientific, Model 1305 U Utility Oven).
2. **Clean distillation tubing.** Loosen pressure fittings and wipe ferrules, fitting nut, and fitting body (Swagelok T-400-6-2) down with a water wet delicate task wipe (Kimberly-Clark Professional, Kimwipes Kimtech Science Brand). With a syringe, pull 0.1 M nitric acid through the PTFE tubing (3.2 mm outer diameter) connecting the bell-shaped quartz tube to the valve (V2 in figure 2, Bürkert 6724) and liquid trap to soak for at least one hour. After the soaking time, repeatedly pass the same nitric acid through the lines, then rinse with multiple aliquots of water until water pH returns to expected value.
3. **Prepare target.** Sand the niobium backing around face of the selected target coin, avoiding contact with the cobalt selenide, then note the target weight. Wipe the entire target capsule with a wet delicate task wipe to remove potential contamination and debris. If working with the ARTMS QIS system, lubricate the o-rings in the shuttle shell and height adapter piece (see supplemental figure 3) with high vacuum grease (Dow Corning), then assemble the height adapter, target coin, water diverter, and target backing into the shuttle shell. If any o-ring appears distressed or damaged, replace them. For the GE Healthcare Solid Target Capsule, replace all o-rings. Assemble the shuttle by placing the target coin in the aluminum backing, centering a 17.5 mm tantalum foil on top, then screwing the shuttle cap in (see supplemental figure 4). The foil should be held in place between the PEEK portion of the shuttle cap and the niobium backing, not contacting the o-ring in the cap. After assembled, both targets system capsules were pressure checked by being placed against an aluminum block with a 6 mm aperture and held together with a ratcheting bar clamp. The compressed air supply (55 psi) was turned on, the gauge given time to stabilize, then the air supply turned off. The gauge is monitored to evaluate the rate of the pressure decrease, with 0.01–0.02 psi decrease per second being satisfactory to maintain vacuum seal within the cyclotron.

Supplemental Figure S3. ARTMS QIS system target shuttle components and construction method. Top (a) photographs of the shuttle shell (face down), water diverter (face down), shuttle backing (face down), diameter adapter (face down), and target cobalt selenide target (face up), listed from left to right, top to bottom. The diameter adapter piece is placed in the shuttle shell (b), then the target coin placed face down on it (c). The water adapted is placed on top of the centered target coin (d), and the backing screwed in to secure everything in place (e). f) Final constructed target from head on (face up).

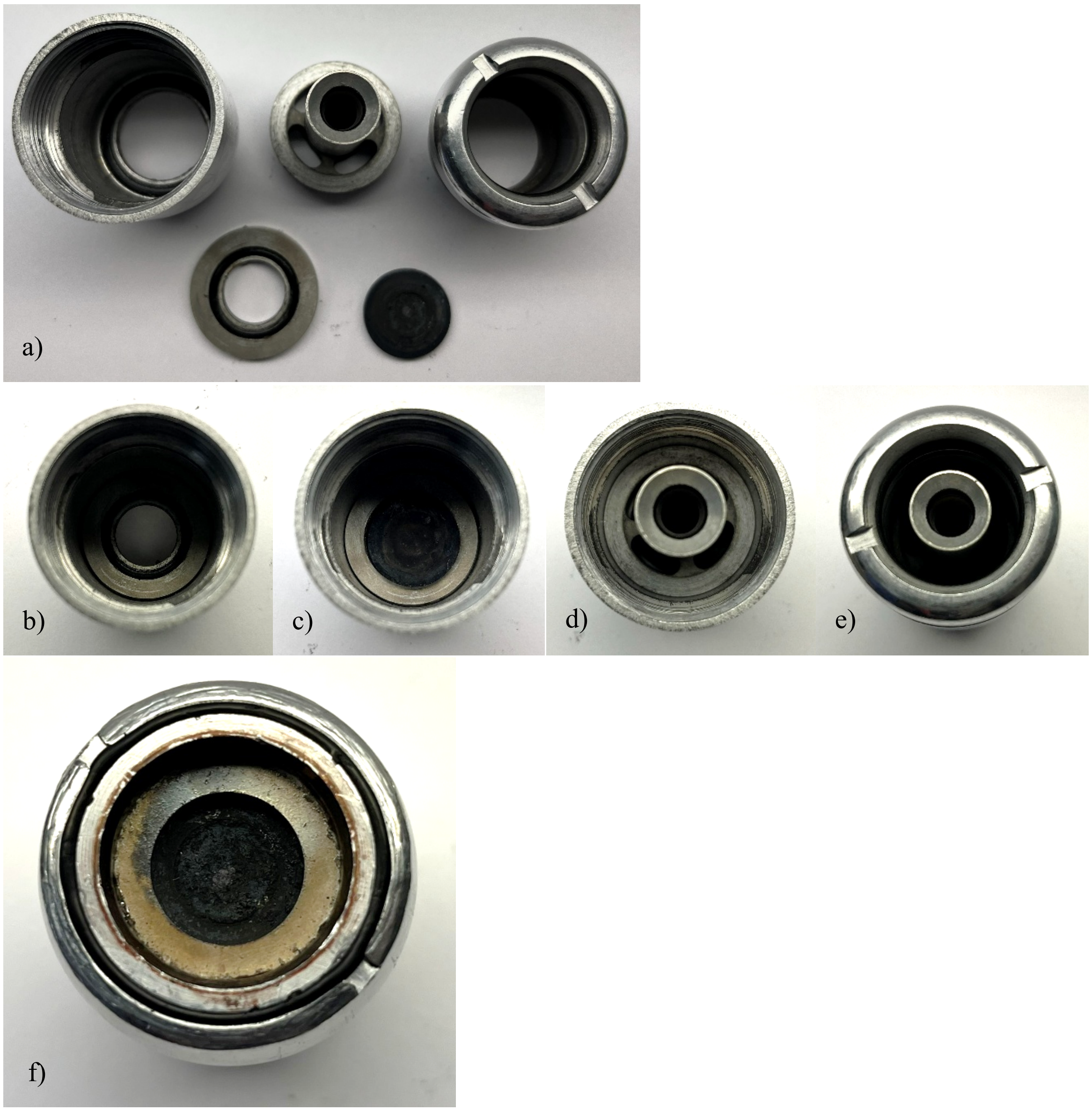

Supplemental Figure S4. GE Healthcare Solid Target System Capsule. a) The aluminum capsule and annular PEEK capsule cap. b) The target coin (face up), and tantalum cover foil disk. c) The completed GE STP target from above.

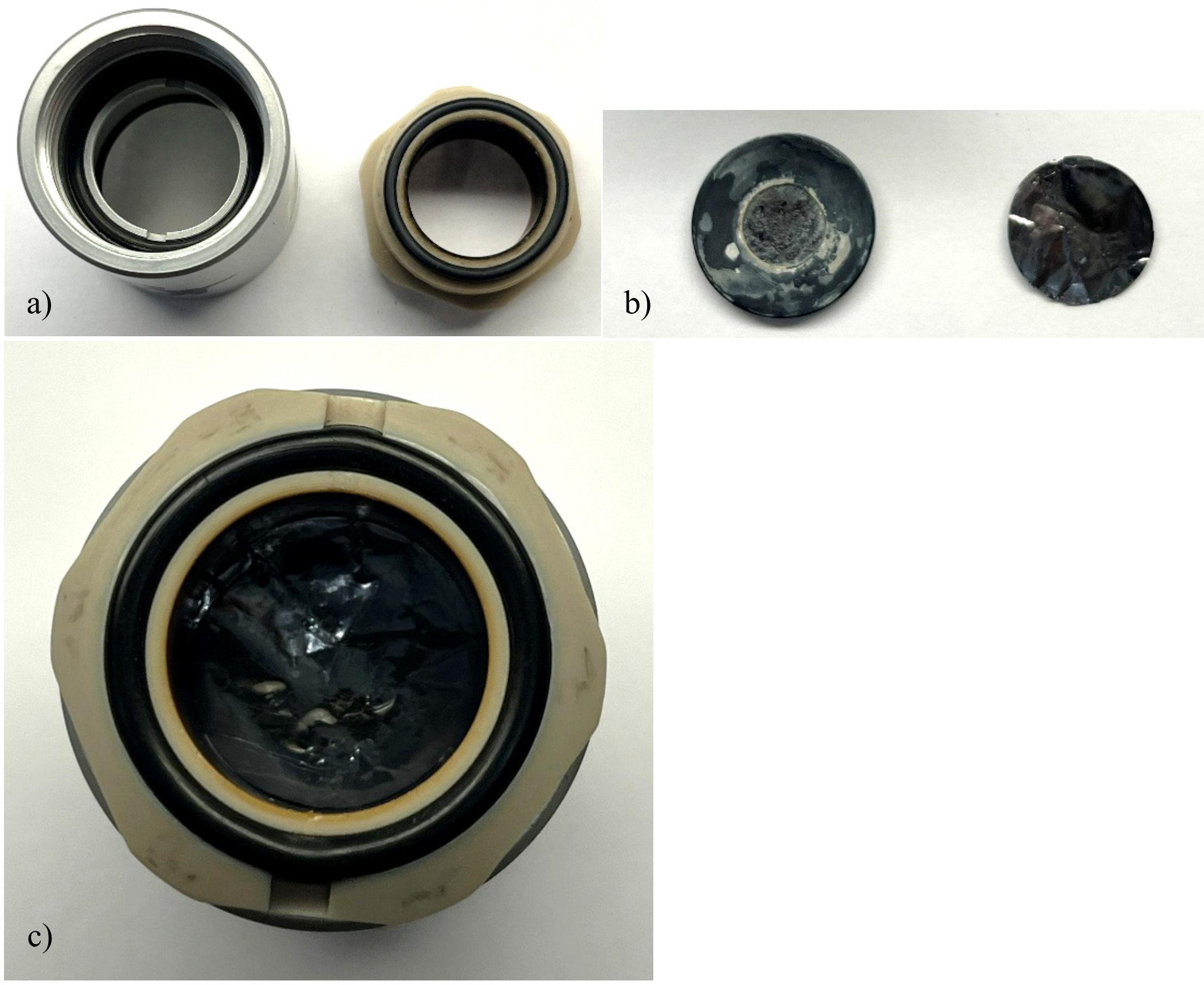

Synthetic and analytical chemistry details

1-(4-chlorophenyl)-8,9-dihydro-2,7,9a-triazabenzo[cd]azulen-6(7H)-one

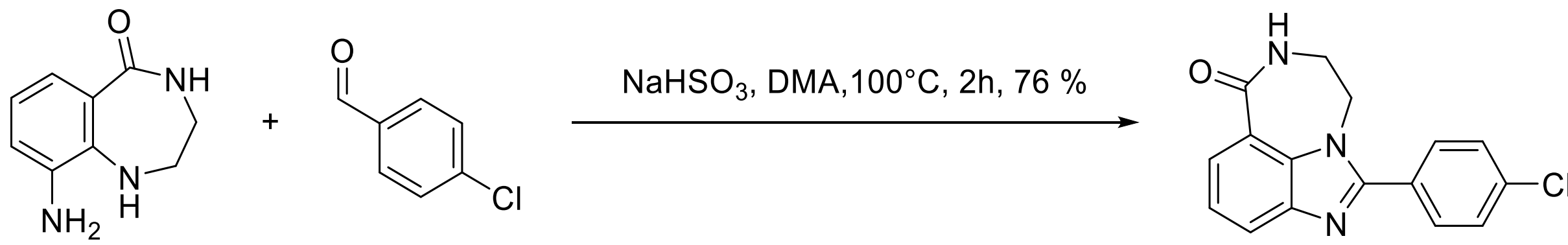

Adapted from methods found in (HF Ridley *et al.*, J. Heterocycl. Chem. *2*(4): 453 – 456, 1965). 9-amino-1,2,3,4-tetrahydro-5H-benzo[e][1,4]diazepin-5-one (150 mg, 0.846 mmol, 1 equiv.), 4-chlorobenzaldehyde (131 mg, 0.931 mmol, 1.1 equiv.) and sodium bisulfite (159 mg, 1.52 mmol, 1.8 equiv.) were suspended in DMA (4 mL) and heated to 100°C for 1.5 h or until the reaction was complete (analyzed by disappearance of the starting material by thin-layer chromatography). The reaction mixture was evaporated directly onto celite and purified by flash chromatography 0-10 % MeOH in EtOAc. The fractions containing product were evaporated and partitioned between water (20 ml) and ethyl acetate (20 ml) to remove remaining DMA. The ethyl acetate was rinsed with brine (20 ml) and dried with Na_2SO_4, filtered and evaporated to give the title product as a pale-yellow solid (191 mg, 0.642 mmol, 76 %) with some solvent impurities. The material was used directly in the next step. Analytical data shown in **Figures S5–S6**.

Supplemental Figure S5. ^{1}H NMR (500 MHz, DMSO) δ 8.4 (t, *J* = 5.8 Hz, 1H), 7.9 – 7.8 (m, 4H), 7.7 – 7.6 (m, 2H), 7.4 (t, *J* = 7.8 Hz, 1H), 4.5 – 4.4 (m, 2H), 3.5 (td, *J* = 5.6, 3.0 Hz, 2H); EtOAc (~5 % n/n imp.) 4.03 (q), 1.99 (s), 1.17 (m); Acetone (~5 % n/n imp.) 2.08 (s); Unknown impurities 1.91 (s), 1.16 (s).

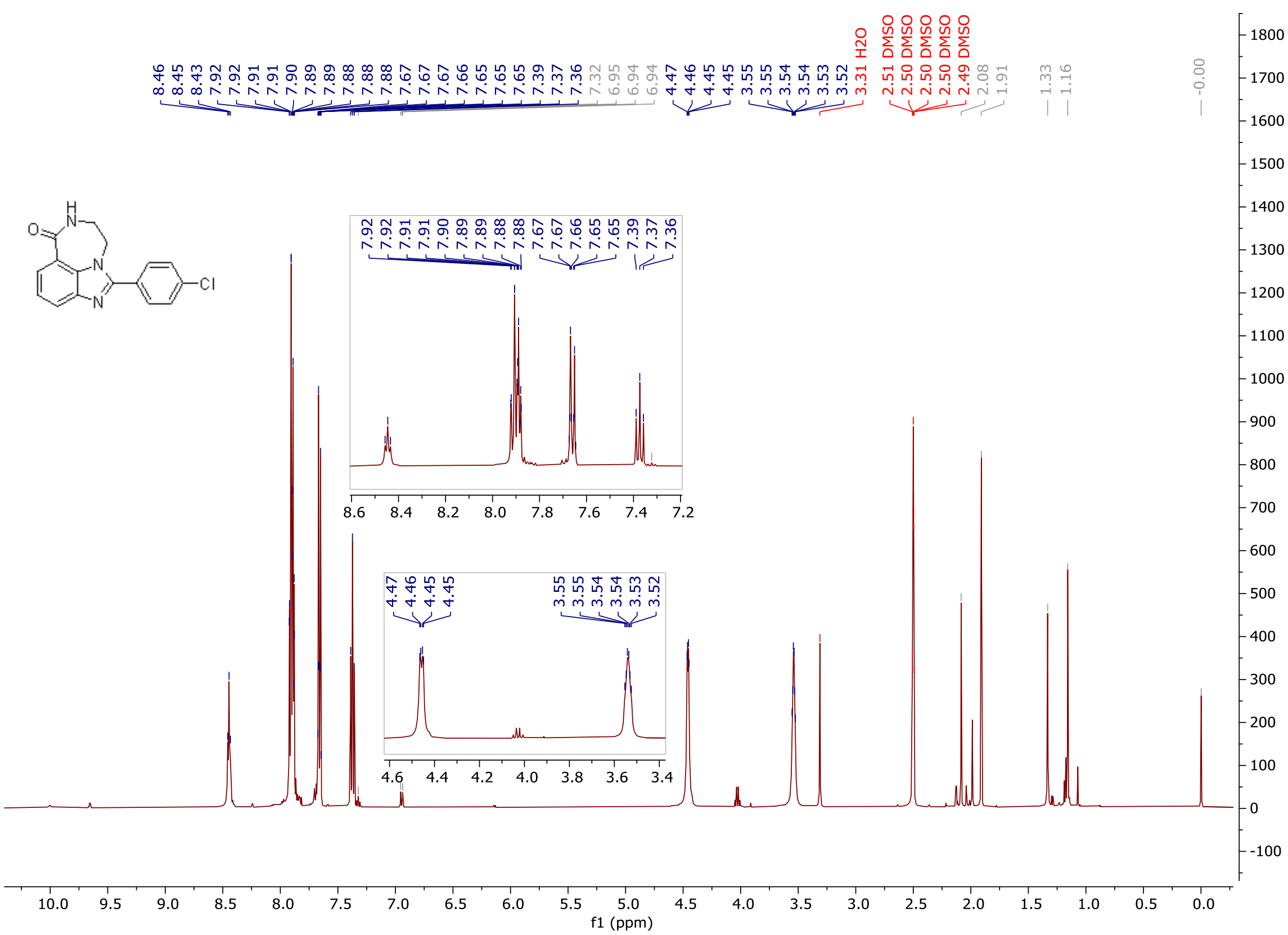

Supplemental Figure S6. ^{13}C NMR (126 MHz, DMSO) δ 167.2, 152.7, 143.2, 134.9, 132.4, 131.4, 128.7, 128.4, 125.6, 123.0, 121.8, 117.9, 50.4, 40.3.

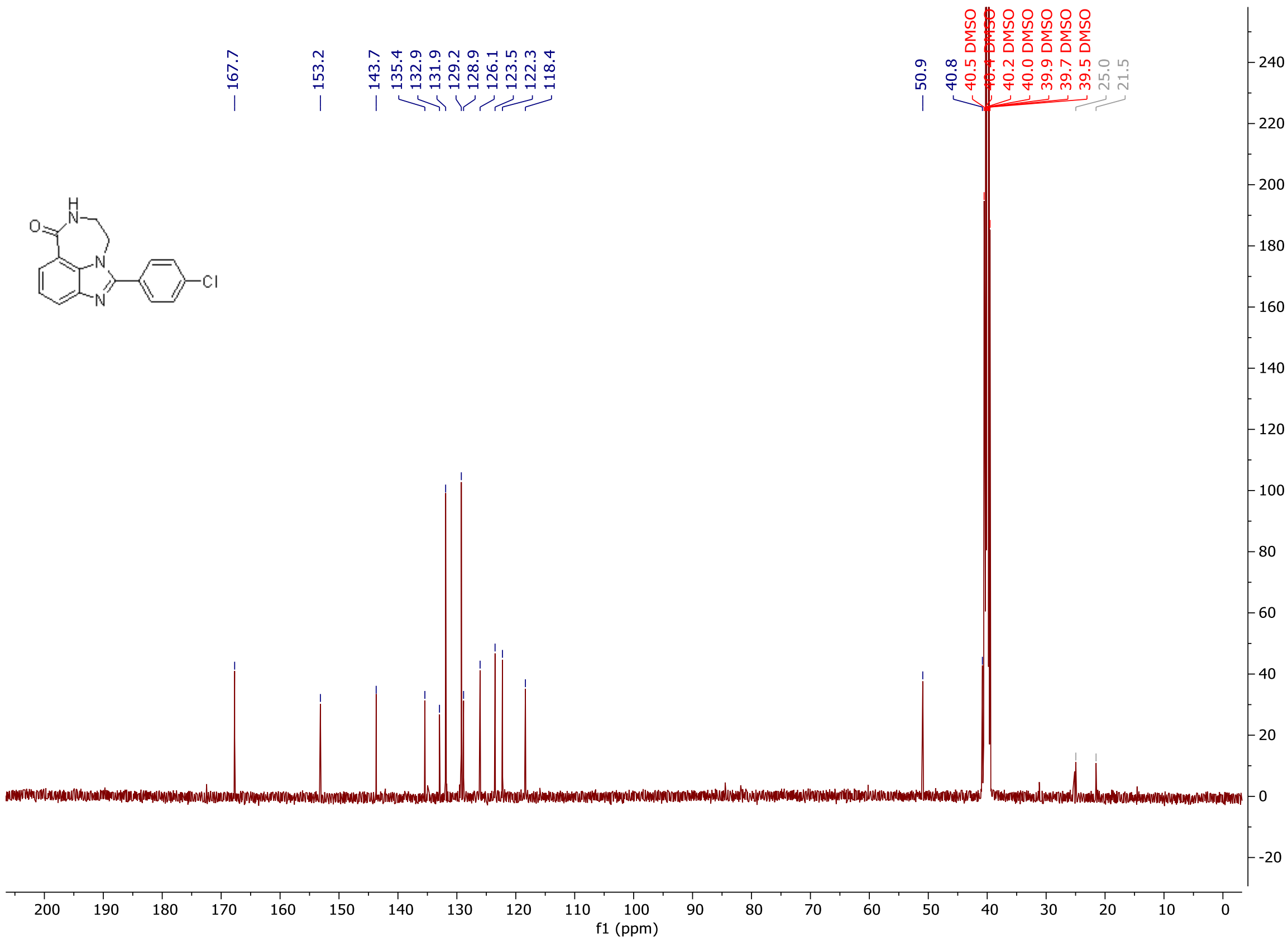

1-(4-(4,4,5,5-tetramethyl-1,3,2-dioxaborolan-2-yl)phenyl)-8,9-dihydro-2,7,9a-triazabenzo[cd]azulen-6(7H)-one (RD1-Bpin)

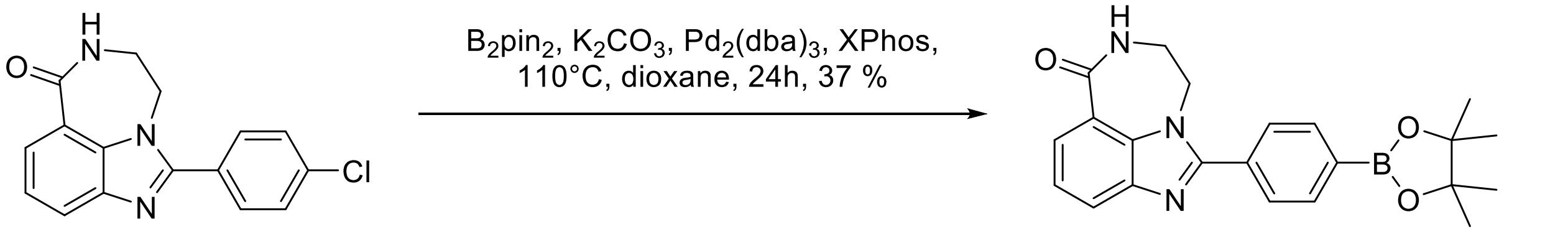

Adapted from methods in (SW Reilly *et al.*, Org. Lett, *20*(7): 1752 – 1755, 2018). 1-(4-chlorophenyl)-8,9-dihydro-2,7,9a-triazabenzo[cd]azulen-6(7H)-one (185 mg, 0.621, 1 equiv), bis(pinacolato)diboron (473 mg, 1.86 mmol, 3 equiv), potassium carbonate (257 mg, 1.86 mmol, 3 equiv), $Pd_2(dba)_3$ (28 mg, 31 µmol, 5 mol%), and XPhos (29 mg, 62 µmol, 10 mol%) were dissolved in dioxane (3 ml, 0.2 M) and heated to 110°C for 5 hours. The reaction mixture was partitioned between EtOAc (25 mL) and water (25 mL) and extracted 3 times with EtOAc (25 mL). The combined organics were washed with brine (25 ml), dried with $MgSO_4$, and evaporated onto celite. The crude mixture was purified by flash chromatography using a gradient of 50-100% EtOAc in hexanes. 1-(4-(4,4,5,5-tetramethyl-1,3,2-dioxaborolan-2-yl)phenyl)-8,9-dihydro-2,7,9a-triazabenzo[cd]azulen-6(7H)-one (RD1-Bpin) was isolated as a pale brown solid (90 mg, 0.231 mmol, 37%) containing ~10% hydroxydeborylated RD1 (RD1-OH). Attempts at further purification led to increased amounts of RD1-OH. Analytical data shown in **Figures S7–S9**.

Supplemental Figure S7. ^{1}H NMR (500 MHz, DMSO) δ 8.4 (t, J = 5.8 Hz, 1H), 7.9 – 7.8 (m, 6H), 7.4 (t, J = 7.8 Hz, 1H), 4.5 (dd, J = 6.4, 2.3 Hz, 2H), 3.5 (q, J = 5.3 Hz, 2H), 1.3 (s, 12H); RD1-OH (~10% n/n imp.) 7.70 (d), 6.94 (d); EtOAc (~4% n/n imp.) 4.03 (q), 1.99 (s), 1.17 (m); Acetone (~4% n/n imp.) 2.08 (s).

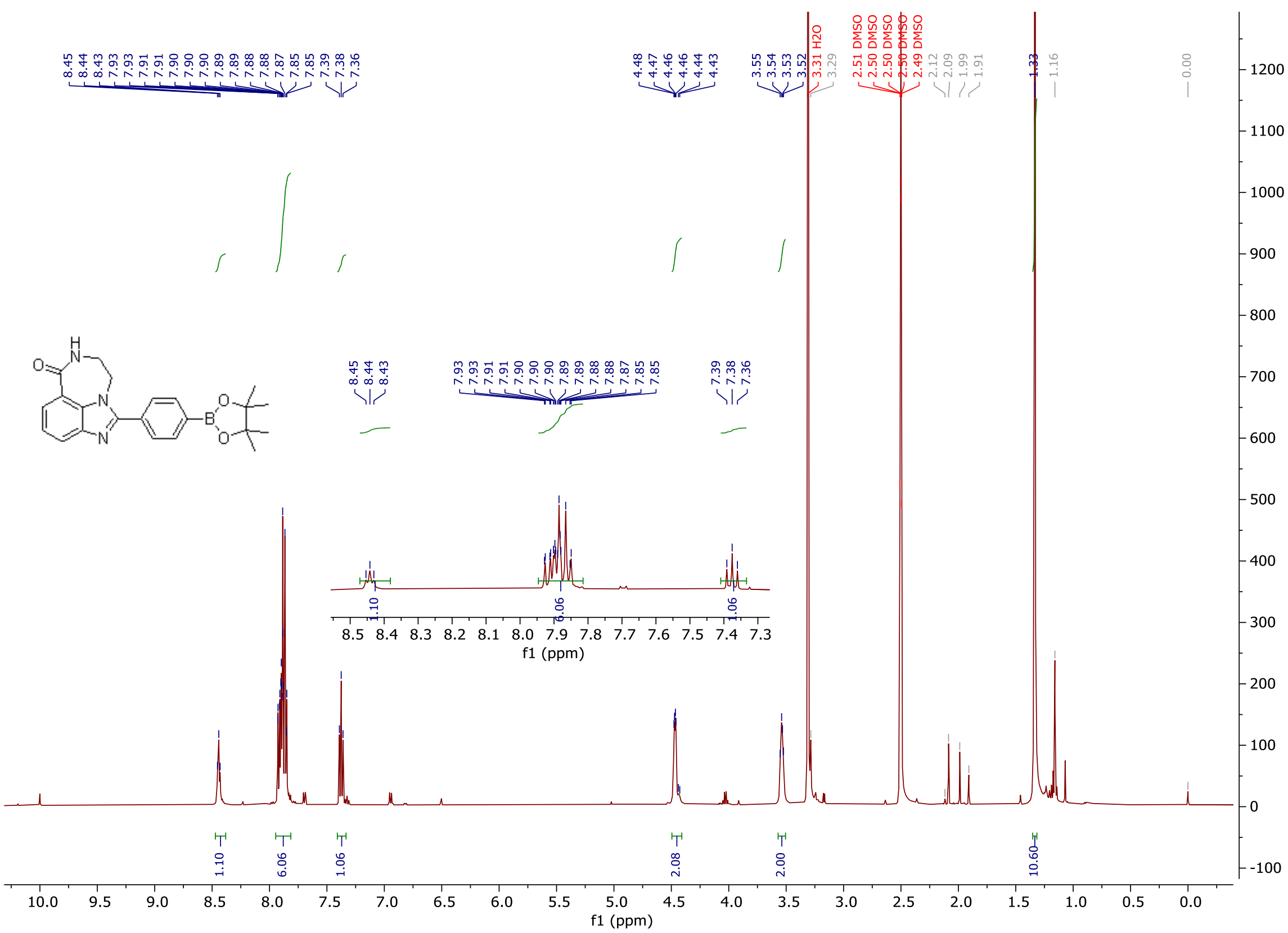

Supplemental Figure S8. ^{13}C NMR (126 MHz, DMSO) δ 167.2, 153.4, 143.3, 134.5, 132.5, 132.1, 129.0, 125.6, 123.0, 121.7, 117.9, 84.0, 50.5, 40.3, 24.7.

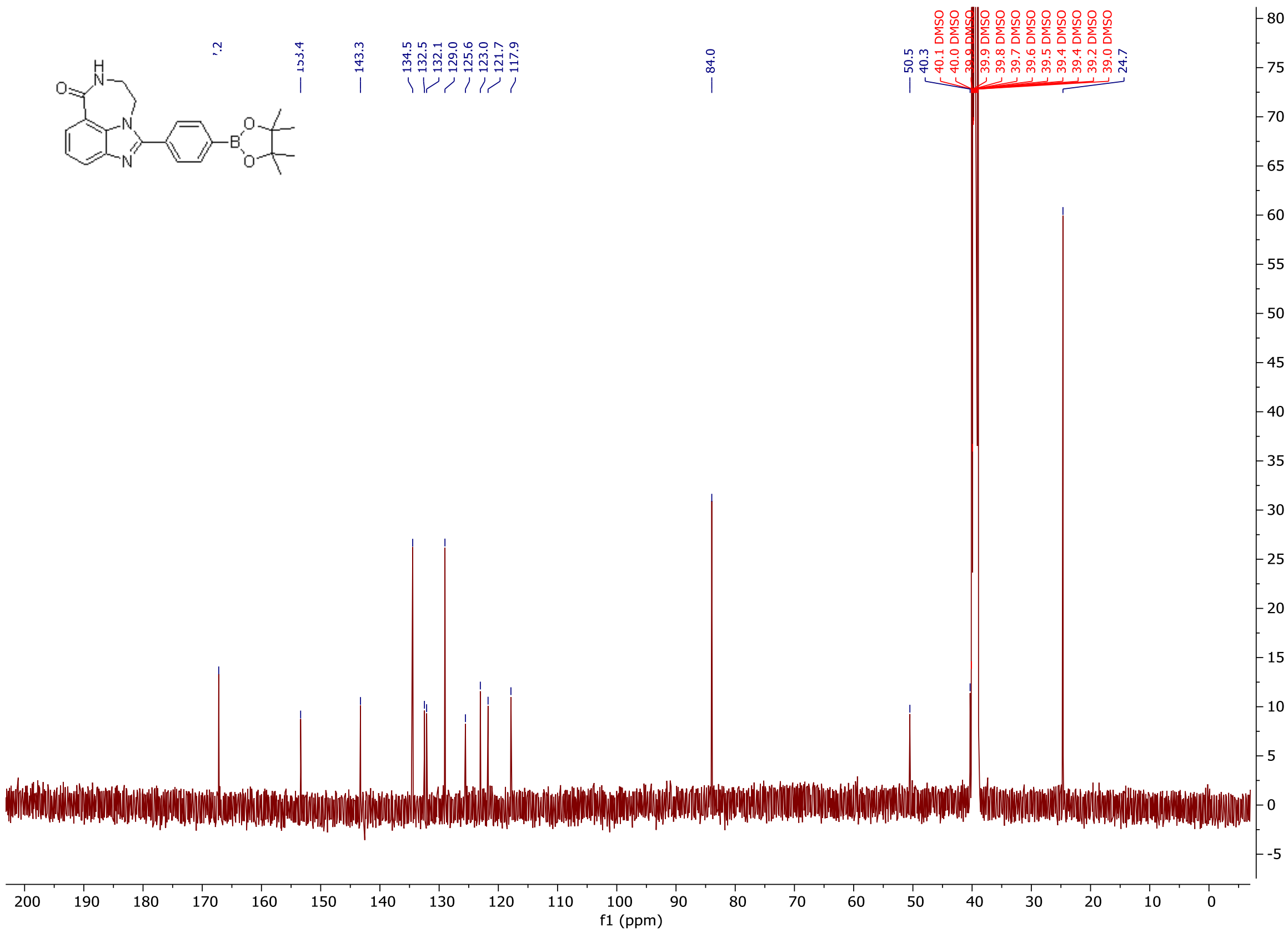

Supplemental Figure S9. HRMS (ESI) m/z: $[M+H]^+$ Calcd. for $C_{22}H_{24}BN_3O_3$+H 390.1984; found 390.1979.

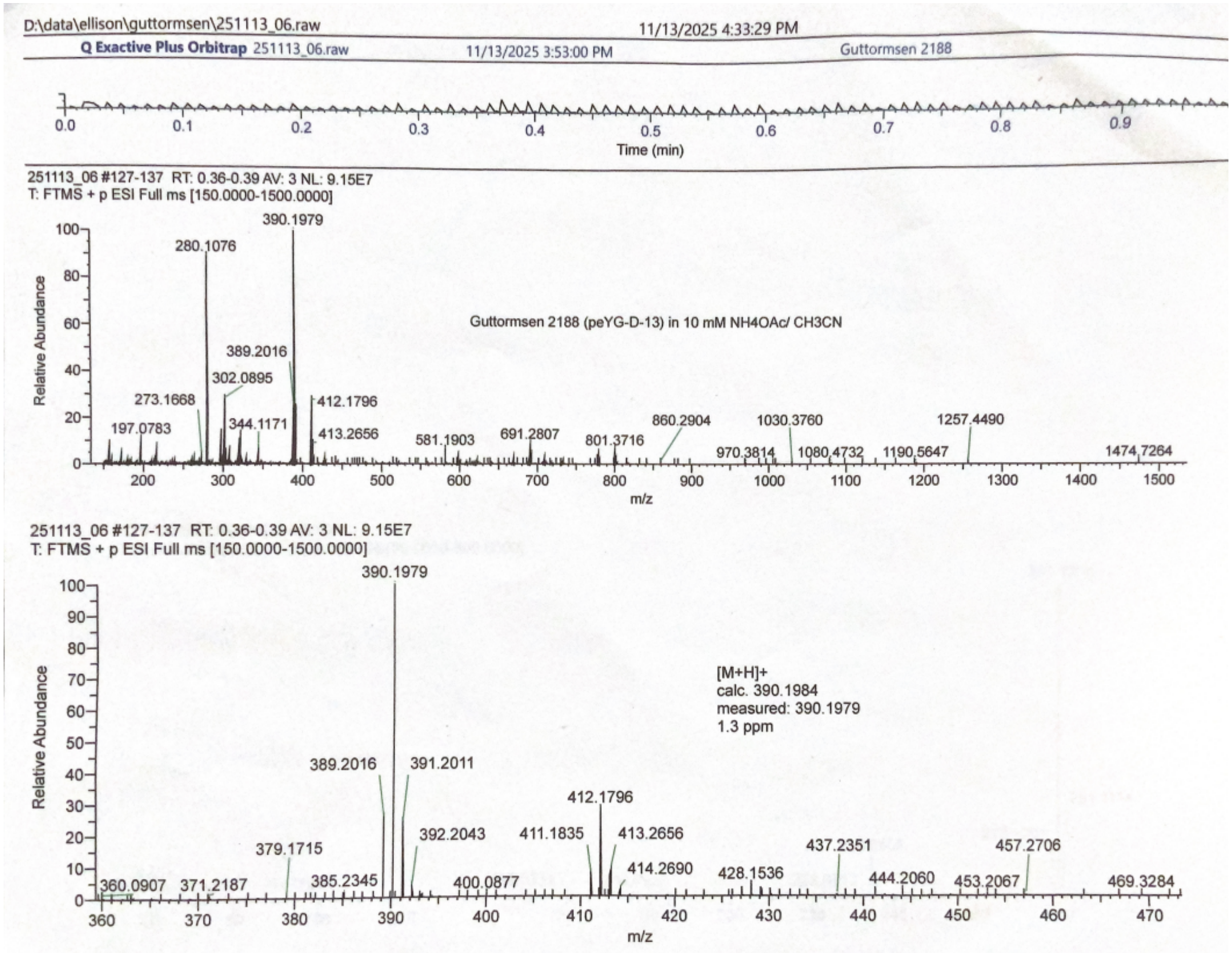

Supplemental Table 2. Analytical HPLC gradient.

Time (min)	0.1 M TEA or 0.1% TFA (%)	Acetonitrile (%)
0	95	5
2	95	5
10	50	50
15	50	50
16	95	5
22	95	5

Molar Activity Determination Methods

Molar activity was determined through analytical HPLC analysis. Before any radioactive analysis, non-radioactive [^{nat}Br]RD1 was dissolved in dimethyl sulfoxide then diluted in 5% acetonitrile/95% water to concentrations of 50, 5, 0.5, and 0.05 µM to serve as standards. These standards, analyzed in triplicate on analytical HPLC, created UV peaks at 8.9 ± 0.2 min post injection with areas linearly proportional to the mass injected. Saline blanks always preceded sample runs. The next sample run was a 0.5 µM or 5.0 µM standard of non-radioactive [^{nat}Br]RD1, followed by an acetonitrile rinse of the injector valve and the radioactive sample.

The 254 nm absorbance peak area of the product was compared to the generated calibration curve. The activity voltage signal was examined to note any radiolytic product breakdown and determine radiochemical purity (RCP, equation 1). The A_m of the product was calculated and recorded using the activity injected, the RCP, and the total moles of product determined from the calibration curve (equation 2).

$$RCP = \frac{RadArea_{PARPi}}{RadArea_{PARPi} + RadArea_{Other}} \quad (1)$$

$$A_m = A_{HB} * \frac{RCP}{\left(mol_{inj} * \frac{Vol_{HB}}{Vol_{inj}}\right)} \quad (2)$$

$RadArea_{PARPi}$ and $RadArea_{Other}$ are the HPLC radiation trace peak areas for the PARP inhibitor and other compounds (including free bromide), respectively. A_{HB} is activity of a holdback of the final reconstituted product. Mol_{inj} is the moles of the PARP inhibitor injected on the column, calculated from the absorbance peak area and the calibration curve. Vol_{HB} and Vol_{inj} are the volumes of the holdback and injected HPLC sample.

Supplemental Figure S10. Radiobromine production yield from cobalt [^{76}Se]selenide (left) and cobalt [^{77}Se]selenide (right) targets as a function of number of previous distillations. A best fit linear regression was applied across targets of different thicknesses, size (19 mm for the ARTMS system, 22 mm for the GE STP), and cobalt selenium inner diameter (9.9 or 11.4 mm) for each isotope, excluding one thick cobalt [^{77}Se]selenide target that did not tolerate the beam well. The y-intercept, corresponding to the first, highest production yield was 53.8 ± 13.9 MBq/µAh for bromine-76, 16.8 ± 4.9 MBq/µAh for most bromine-77 targets, and 22.2 ± 2.7 MBq/µAh for the thick bromine-77 target. The fit slope corresponds to the loss of activity production after each distillation. When dividing the slope by the y-intercept, the fractional yield loss was calculated: 2 ± 8% for bromine-76, 2 ± 3% for most bromine-77, and 10 ± 3% for the thick bromine-77 target. The difference in slope between the thick target and the average of the rest of the bromine-77 targets is statistically significant. The two production yields much lower than the trendline for bromine-76 are likely due to total activity quantification errors.

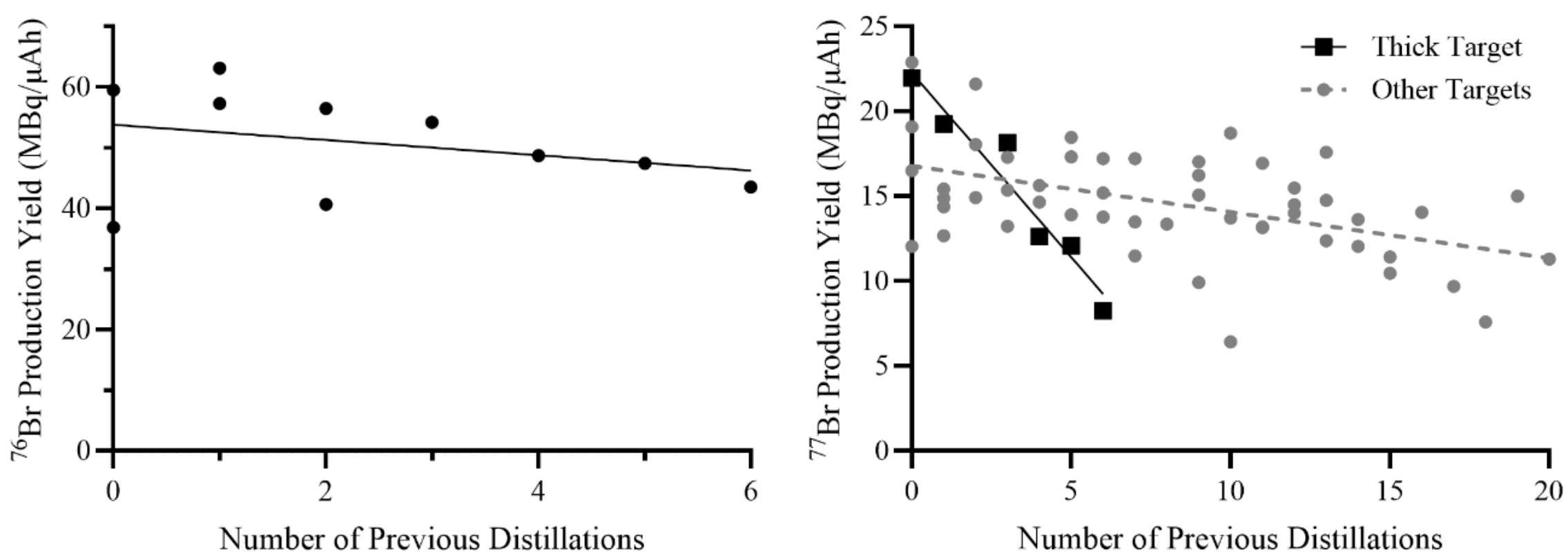

Supplemental Figure S11. Recorded radiation signals during the distillation and subsequent rinsing of the glassware, normalized to the maximum detected signal at each detector for this distillation. The gradual drop in furnace signal from 1 to 510 seconds shows release of radiobromide from the target, with the high baseline caused by the decay of other radioisotopes remaining in the target, chiefly molybdenum-93m ($t_{1/2}$ = 6.85 h). The sudden drop in the furnace signal occurred when the target and quartz were removed from detector line of sight for cooling. Likewise, the sharp rise in the liquid trap signal corresponds to radiobromine arrival into the liquid trap after the first rinse, carrying with it was majority of the recoverable activity.

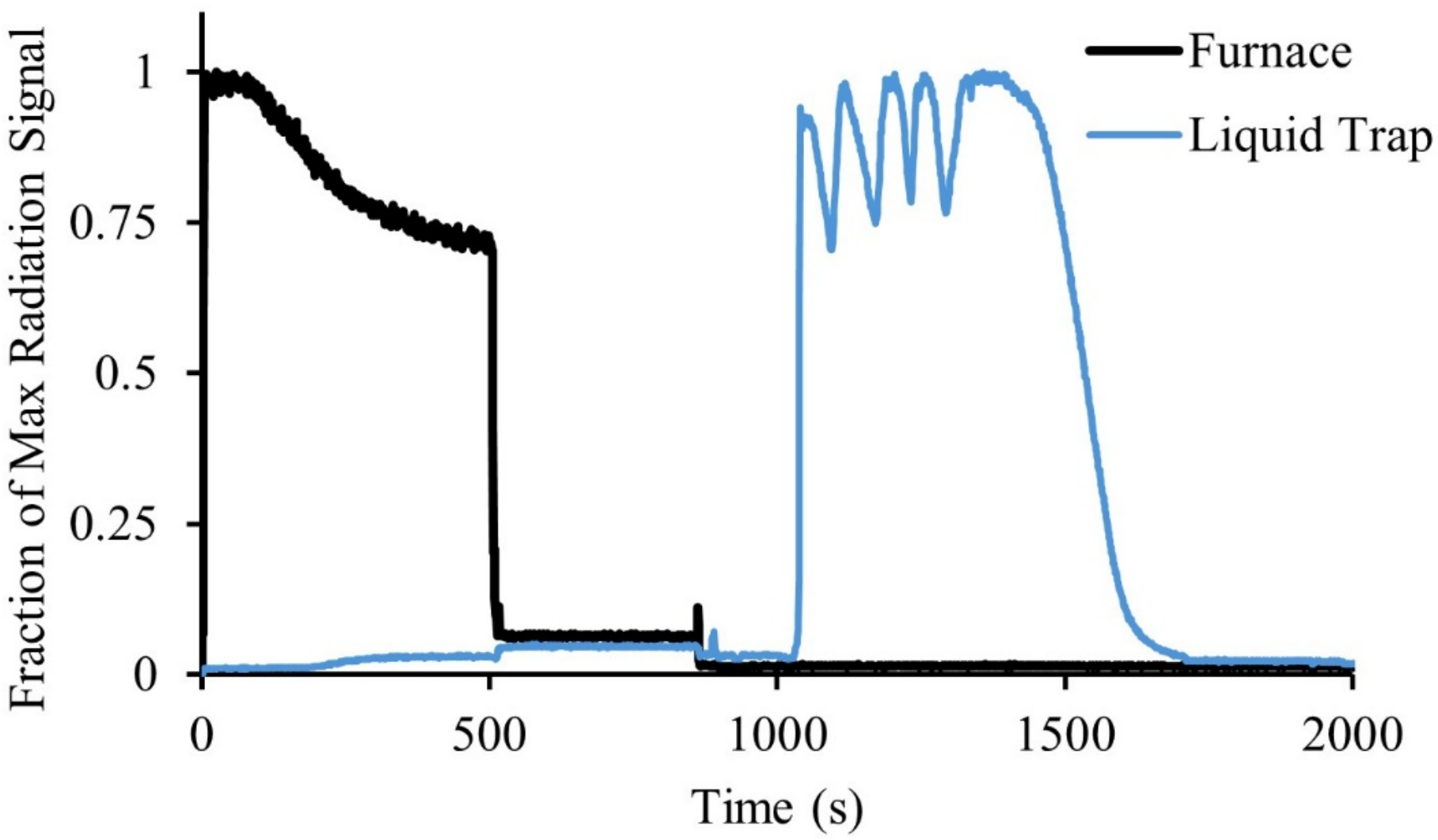

Supplemental Figure S12. (a) Analytical HPLC chromatograms of 254 UV absorbance (black line) for 50 μM [^{nat}Br]RD1 (top), a PBS blank (middle), and [^{77}Br]RD1 (bottom), with the radiation trace (red line) of [^{77}Br]RD1 in the bottom plot as well. Because the injection of [^{77}Br]RD1 contained no [^{nat}Br]RD1, the absorbance peak was small, but it aligned with the radiation signal and the 50 μM [^{nat}Br]RD1 standard elution peak, with no other distinct UV peaks that were not also present in the PBS blank. (b) The preparative HPLC chromatograms of the [^{77}Br]RD1 analyzed in (a) shows typical product separation from other products and contaminants. The dotted line represents the time at which the preparative HPLC mobile phase switched from 30% acetonitrile/70% 0.1 M ammonium formate to 65% acetonitrile/35% water. The red band in both figures represents the expected elution time of [^{77}Br]RD1.

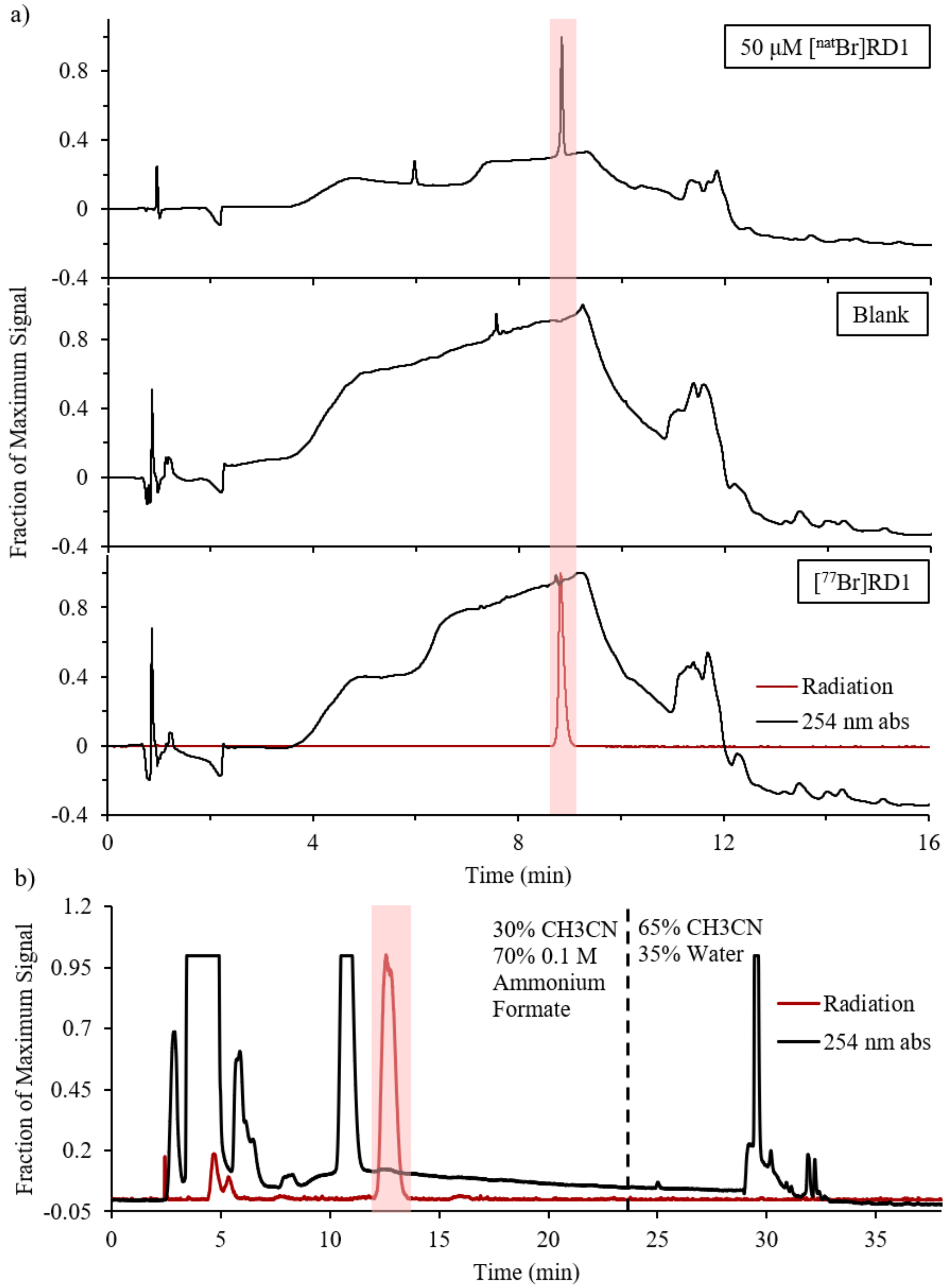

Supplemental Figure S13. Molar activity of [^{76}Br]RD1 (top) or [^{77}Br]RD1 (bottom) as a function of activity in the final formulation of the radiopharmaceutical. The molar activity was likely proportional to the final activity because of contamination from some non-radiobrominated form of RD1 (such as hydroxylated RD1). This contaminant could have formed during the reaction with unreacted BPin, then because of similar electronegativity of the group to bromine, had an identical retention time on both the preparative and analytical HPLC C18 columns.

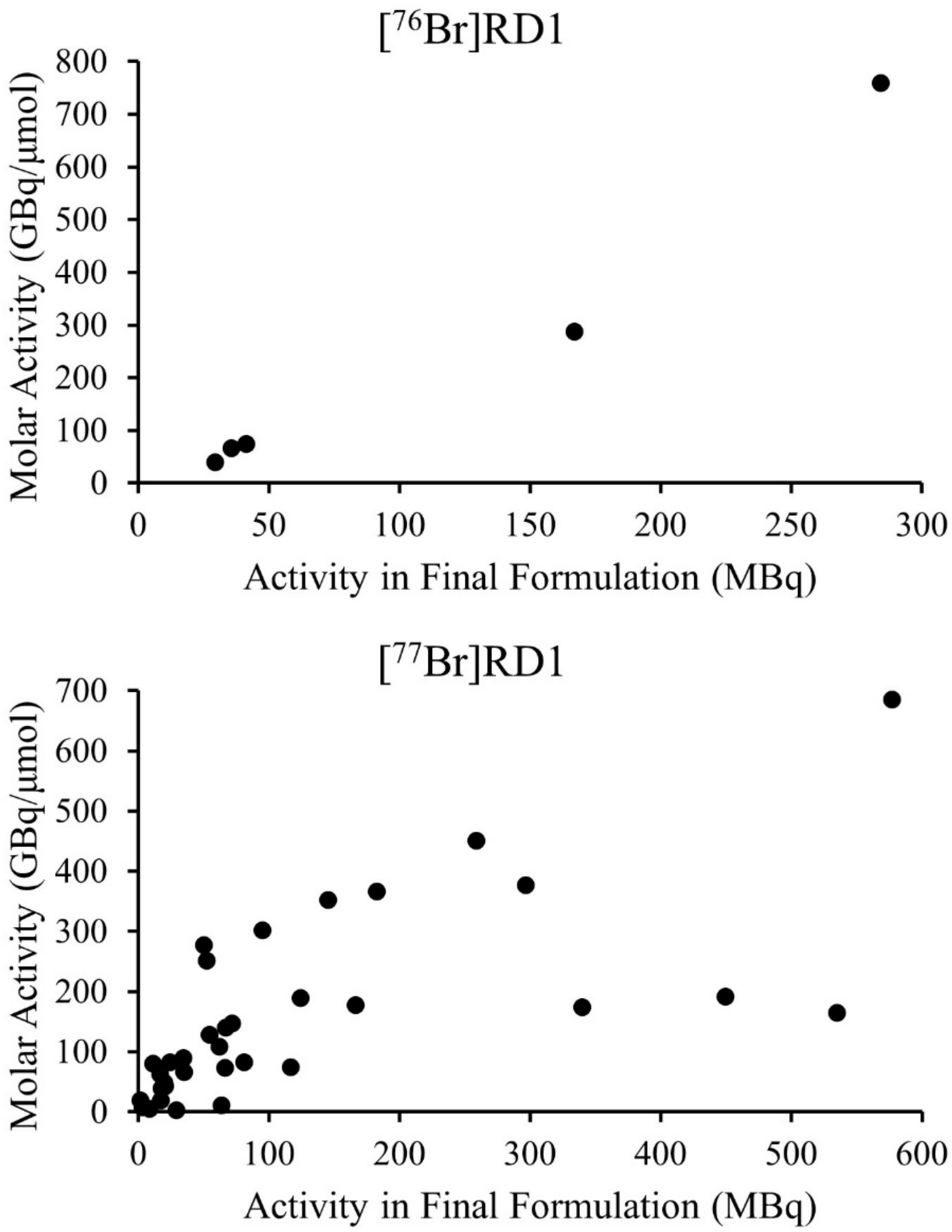

Supplement: Supplementary file 1 [file 41181_2026_458_MOESM1_ESM.pdf]
